# Target deubiquitinase OTUB1 as a therapeatic strategy for BLCA via β-catenin/necroptosis signal pathway

**DOI:** 10.7150/ijbs.94013

**Published:** 2024-07-02

**Authors:** Yihao Liao, Jiaming Liang, Youzhi Wang, An Li, Wenbo Liu, Boqiang Zhong, Keke Wang, Diansheng Zhou, Tao Guo, Jianing Guo, Xi Yu, Ning Jiang

**Affiliations:** 1 Department of Urology, Tianjin Institute of Urology, The Second Hospital of Tianjin Medical University, Tianjin 300211, China.; 2 Department of Urology, The First College of Clinical Medical Science, China Gorges University & Yichang Central People's Hospital, Yichang, Hubei, 443003, 100000, China.; 3 Department of respiratory medicine, Chinese PLA general hospital, Beijing, China.; 4 Department of Pathology, The Second Hospital of Tianjin Medical University, Tianjin 300211, China.; 5 Department of Anesthesia, The Second Hospital of Tianjin Medical University, Tianjin 300211, China.

**Keywords:** OTUB1, deubiquitination, β-catenin, BLCA, chemoresistance

## Abstract

Ubiquitination, a prevalent and highly dynamic reversible post-translational modification, is tightly regulated by the deubiquitinating enzymes (DUBs) superfamily. Among them, OTU Domain-Containing Ubiquitin Aldehyde-Binding Protein 1 (OTUB1) stands out as a critical member of the OTU deubiquitinating family, playing a pivotal role as a tumor regulator across various cancers. However, its specific involvement in BLCA (BLCA) and its clinical significance have remained ambiguous. This study aimed to elucidate the biofunctions of OTUB1 in BLCA and its implications for clinical prognosis. Our investigation revealed heightened OTUB1 expression in BLCA, correlating with unfavorable clinical outcomes. Through *in vivo* and *in vitro* experiments, we demonstrated that increased OTUB1 levels promote BLCA tumorigenesis and progression, along with conferring resistance to cisplatin treatment. Notably, we established a comprehensive network involving OTUB1, β-catenin, necroptosis, and BLCA, delineating their regulatory interplay. Mechanistically, we uncovered that OTUB1 exerts its influence by deubiquitinating and stabilizing β-catenin, leading to its nuclear translocation. Subsequently, nuclear β-catenin enhances the transcriptional activity of c-myc and cyclin D1 while suppressing the expression of RIPK3 and MLKL, thereby fostering BLCA progression and cisplatin resistance. Importantly, our clinical data suggest that the OTUB1/β-catenin/RIPK3/MLKL axis holds promise as a potential biomarker for BLCA.

## Introduction

BLCA (BLCA) stands out as one of the most prevalent malignant cancers globally. Recent statistics from America in 2023 underscore its significant impact, with approximately 81,180 newly diagnosed BLCA patients. Among these cases, males represent around three-quarters, while females comprise the remaining one-quarter. Tragically, BLCA-related reasons contribute to 17,100 deaths, with 12,120 being male patients and 4,980 female patients. Further analysis reveals that BLCA ranks fourth in terms of morbidity and eighth in mortality among all cancer types affecting males^1^. Recently, most patients obtained relatively long and free-progression survival time with the rapid development of treatment measures and strategies, including total/partial cystectomy^2^ coupled with intravesical infusion therapy^3^, targeted therapy^4^, endocrine therapy, etc. 5. Currently, the biggest challenge in BLCA treatment is high recurrence rate after surgery and chemotherapy^6^. Therefore, it's imperative to recognize that comprehending the pathophysiological mechanisms underlying BLCA carcinogenesis holds equal importance alongside comprehensive treatment strategies. Protein ubiquitination degradation and the deubiquitination enzyme system are recognized for their involvement in numerous pathophysiological and biological processes that regulate proliferation and carcinogenesis^7^, ^8^. Numerous members have been identified as key players in the onset and advancement of various cancers by mediating the degradation and stability of critical proteins. Among these, OTU Domain-Containing Ubiquitin Aldehyde-Binding Protein 1 (OTUB1), a member of the OTU deubiquitination family, has emerged as a regulator influencing the development and progression of multiple cancers and immune-related disorders through various signaling pathways^9^. Recent studies showed that OTUB1 is elevated in colorectal cancer, promotes metastasis and serves as a marker of poor prognosis of colorectal cancer^10^. In previous study, we found that OTUB1 facilitates the progression and proliferation of prostate cancer via deubiquitinating and stabilizing CyclinE1 depending on its deubiquitination activity^11^. Moreover, more roles of OTUB1 in other cancers also are identified, for example, in breast cancer^12^, esophageal cancer^13^, and lung cancer^14^, the sophisticated role in multiple diseases further implies its critical position. Although several studies have shown that OTUB1 regulates the progression of BLCA via mediating ATF6^15^ or E2F1^16^ stability, the pathophysiological mechanism is incomplete, OTUB1 functions remain much to excavate and figure out.

## Material and methods

### Patient samples and information

In our study, we collected 42 paired BLCA tissues and para-cancer tissues from cystectomy specimens without radiation therapy, and chemotherapy in the department of urology, The second hospital of Tianjin medical university (Tianjin, China). These tissues were further diagnosed by a professional doctor from the department of pathology. We collected paired tissues according to the strict screening criteria, these patients who underwent total cystectomy without radiation therapy, and chemotherapy was selected for this study; while these patients who underwent electro-cystectomy, puncture or radiation therapy, or chemotherapy were excluded. The collecting tissue processes were approved by the ethics committee of the second hospital of Tianjin medical university and complied with the Helsinki Declaration of Human Rights.

### Antibodies information

In western blot, immunohistochemical staining, immunofluorescence staining, co-immunoprecipitation and other assay, multiple related antibodies were needed: anti-OTUB1: affinity, DF9998, Rabbit, western blot (1:1000), immunohistochemical staining (1:150), immunofluorescence staining (1:100); anti-OTUB1: abcam, ab175200, co-immunoprecipitation (1:20); anti-GAPDH: affinity, T0004, western blot(1:1000); anti-beta actin: affinity, T0022, western blot (1:1000); anti-tubulin alpha: affinity, AF0524, western blot (1:1000); anti-β-catenin: affinity, AF6266, western blot (1:1000), immunohistochemical staining (1:150), immunofluorescence staining (1:100); anti-CDK4: affinity, DF6102, western blot (1:1000); anti-cyclin D1: affinity, DF6386, western blot (1:1000); anti-AXIN-2: affinity, DF6978, western blot (1:1000); anti-TCF1: CST, C63D9, western blot (1:1000); anti-c-myc: affinity, AF6054, western blot (1:1000); anti-ki67: ZSGB-BIO, ZA-0502, IHC (working fluid); anti-Ubiquitin: affinity, AF0289, western blot (1:1000); anti-Flag: affinity, T0053, western blot (1:1000), co-immunoprecipitation(1:20); anti-MDR1: affinity, AF5185, western blot (1:1000); anti-BCRP: affinity, AF5177, western blot (1:1000); anti-YB-1: affinity, AF7832, western blot (1:1000); anti-RIPK3: affinity, DF10141, western blot (1:1000); anti-MLKL: affinity, DF7412, western blot (1:1000); anti-MLKL (ser 345): abcam, ab196436, western blot (1:1000).

### Cell lines and culture

Human BLCA cell line (T24, 5637, BIU-87, EJ, 253J-BV) and human bladder epithelial immortalized cell (SV-HUC) were purchased from ATCC cell bank (America). T24, 5637, BIU-87, EJ and 253J-BV cells were cultured with RPMI 1640 medium supplemented with 10% fetal bovine serum and 1% penicillin/streptomycin in a humidified environment containing 5% CO2 at 37◦C; while SV-HUC was cultured with F-12K medium supplemented with 10% fetal bovine serum and 1% penicillin/streptomycin in the cell incubator containing 5% CO2 at 37◦C.

### Transfection and inhibitor treatment

In this study, we transfected selected cells with multiple siRNA, shRNA lentivirus and plasmids by using the transfection reagent LipofectamineTM 2000 (Invitrogen) according to the protocol. Firstly, we planted 5×10^5^ cells into 6-well plates and transfected with 3ug plasmids or 100 nM siRNA after 24 hours. These transfected cells were harvested for subsequent experiments 48 hours after transfection, containing western blot, transwell assay, MTT assay and other experiments.

XAV-939 (5uM, 10uM) was a β-catenin-specific inhibitor and obtained from MCE (HY-15147), which was further diluted into different concentrations for subsequent experiments.

### Bioinformatics and data extracting

We downloaded the BLCA dataset in TCGA and screened the differentially expressed genes (DEGs). The mRNA expression profile data and clinical information data on BLCA (BLCA) were downloaded from the TCGA database (https://www.cancer.gov/about-nci/organization/ccg/research/structural-genomics/tcga), and the differential expression between normal tissues and BLCA tissues was analyzed by R's DESeq2 software package. The differential expressed genes between normal tissue and BLCA tissue were selected(|log2FoldChange|>1 & padj<0.05). Based on BLCA information in the TCGA database, the deubiquitinase OTU superfamily members were differentially expressed in normal tissue and BLCA tissue. OTUB1 expression in normal tissue and BLCA tissue was further analyzed in the Box Scatter. In all differential expressed genes in normal tissue and BLCA tissue, 5859 up-regulated genes (Table S1) and 3022 down-regulated genes (Table S2) are listed in the supplementary material. The relationship between OTUB1 and β-catenin was predicted via the online GEPIA database (http://gepia.cancer-pku.cn/detail.php?gene=OTUB1). And the OTUB1 protein expression levels in BLCA tissue and normal tissue were obtained from the Human Protein Atlas online database (https://www.proteinatlas.org/search/OTUB1); and β-catenin expression also was found (https://www.proteinatlas.org/ENSG00000168036-CTNNB1). The complex network association between OTUB1 and β-catenin was found in the GENEMANIA online database (http://genemania.org/search/homo-sapiens/OTUB1/CTNNB1/).

### Immunohistochemical staining

All 42 paired BLCA and para-cancer tissue were obtained from cystectomy in the department of urology, the second hospital of Tianjin medical university; the mice bladders in BNN-induced-BLCA mice and subcutaneous tumors in nude mice were removed in a sterile environment. All collected human BLCA samples, mice bladders and subcutaneous tumors were preserved in formalin, which further was embedded with paraffin and sliced with 4um thickness. These slices were processed in the following order according to the protocol: xylene dewaxing; hydration at different concentrations of alcohol; antigen repair with peroxide; antibody Incubation; rewarming; secondary antibody incubation; DAB staining; hematoxylin-stained nuclei; dehydration at different concentrations of alcohol; xylene clear; the slices were sealed with neutral glue. Finally, the expression of OTUB1, Ki-67, and β-catenin was observed and analyzed in under a Zeiss microscope (100×, 200×).

### Immunofluorescence staining

We planted 1.5*10^4^ EJ cells in 24-well plates and cells were grown on Labtek II-CC2 chamber slides (Nunc). After 24 hours, we washed slides and foxed cells with 4% paraformaldehyde for 5min at 4°C, further washed with phosphate buffer saline (PBS) and blocking buffer (PBS with 5% BSA and 0.1% Triton X-100), incubated with primary antibody (OTUB1: affinity, DF9998, 1:100; β-catenin: affinity, AF6266, 1:100) overnight at 4°C. On the next day, we added sequentially secondary antibodies (Invitrogen) and DAPI (nuclear staining; Sigma). Finally, the location of OTUB1 and β-catenin in a cell were presented in the Fluorescence Imaging System.

### Western blot and co-immunoprecipitation

The total protein of cells, clinical samples, and mice tumors were extracted by using a mixture of RIPA lysate and PMSF (RIPA: PMSF=100:1), and protein concentration was further determined by using a BCA kit. We separated the protein samples by 10% SDS-polyacrylamide gel electrophoresis (PAGE) and further transferred protein to the polyvinylidene difluoride (PVDF) membrane by electric transfer. The transferred PVDF membrane was further washed with TBST wash buffer and blocked with 5% skim milk powder TBST buffer, finally incubated with the above-mentioned western blot-related antibodies overnight at 4°C. Next day, the membrane was washed and incubated with anti-rabbit/mouse IgG secondary antibodies at room temperature. The specific bands were detected by ECL chemiluminescence liquid and Luminescent Imaging Workstation, and the intensity and expression of the protein were further measured and analyzed by using the software ImageJ and GraphPad Prism 8. In the co-immunoprecipitation assay, the extracted protein with RIPA lysate and PMSF was enriched by incubating with relative antibody liking OTUB1, β-catenin, Flag and other antibodies overnight at 4°C, then the protein was further purified according to the protocol. Finally, the corresponding antibody-enriched protein was collected and further mixed with loading buffer, which was handled following the steps of western blot.

### RT-PCR

The total RNA of different cell and clinical samples was extracted by using TRIzol reagent according to the protocol. Quantitative total RNA was further reverse transcribed into cDNA by Reverse Transcription Kit, obtained cDNA as a template for further amplification conduct RT-PCR to measure related gene expression. GAPDH was used as the normalized control.

### MTT assay

We transfect T24, EJ cells with siOTUB1, shOTUB1 lentivirus, differently designed plasmids for 48 hours, or treated cells with β-catenin inhibitor XAV-939 in different concentration gradients for 24 hours. We planted approximately 2.5*10^3^ cells into per well in a 96-well plate and cultured for another 1-4 days at 37°C. Then, 30ul MTT reagent was added into per well at a fixed time every day, the 96-well plate was incubated for 2 hours at 37°C. Finally, the MTT reagent was removed and 150ul DMSO was added to each well for 15min incubation at 37°C, the number of cells was measured by the absorbance at 490nm with a microplate reader. Software GraphPad Prism 8 was used for statistical analysis.

### Transwell and migration

We transfect T24, EJ cells with siOTUB1, shOTUB1 lentivirus, differently designed plasmids for 48 hours, or treated cells with β-catenin inhibitor XAV-939 in different concentration gradients for 24 hours. We suspended 2*10^4^ cells with 200ul 1640 culture medium and planted them into the top chamber of a 24-well-transwell-plate (Corning, 8 m pore size), and 800ul 1640 containing 10% FBS was added to the bottom chamber. The 24-well-transwell-plate was incubated for another 48 hours at 37°C, the chamber was washed with PBS for 3min, fixed with 4% paraformaldehyde for 25min at 4°C and stained with crystal violet for 20min at 4°C. These migrated cells in the chamber were observed and calculated under an optical microscope, which was further analyzed by the software ImageJ and GraphPad Prism 8.

### Cell cycle assay

EJ cells were transfected with shOTUB1 lentivirus and collected at the exponential growth phase, approximately 1*10^5^ cells were calculated and incubated with 70% alcohol overnight at 4°C to fix cells. Then, cells were washed with pre-cooled PBS repeatedly to remove residual alcohol, propidium iodide was used for cell staining. Finally, cells were separated into individual discrete cells, cell cycle distribution was detected and monitored with a FACScan flow cytometer (BD Biosciences). The results of cell cycle were further analyzed by FlowJo and GraphPad Prism 8 software.

### Clone formation assay

We transfect T24, EJ cells with siOTUB1, shOTUB1 lentivirus, differently designed plasmids for 48 hours, or treated cells with β-catenin inhibitor XAV-939 in different concentration gradients for 24 hours. Total 2*10^3^ cells were planted into the 6-well plate and continued to culture for another week in 1640 containing 10% FBS at 37°C. Cells in 6-well plates were washed with PBS for 3min twice, fixed with 4% paraformaldehyde for 25min at 4°C and stained with crystal violet solution for 20min at 4°C. Then, the plate was washed again and air drying and the number of the clone was observed and calculated by the software ImageJ.

### Wound healing assay

We transfect T24, EJ cells with siOTUB1, shOTUB1 lentivirus, differently designed plasmids, or treated cells with β-catenin inhibitor XAV-939 in different concentration gradients. 24 hours after transfecting or before XAV-939 treatment, we drew a channel on the monolayer cells with a 10ul micropipette tip, washed cells with PBS and photographed them as the control images. The plate was cultured for another 24 hours at the cell incubator, and cells were washed with PBS again and photographed as the 24h images. The changes in cell migrating distance were measured to compare the effects of intervening factors on proliferation ability.

### RNA-seq analysis

BLCA EJ cells were transfected with shOTUB1 lentivirus and purified with 4ug/ml puromycin 1640 culture medium containing 10% FBS. The stably expressed cells were prepared for RNA sequencing, which was supported by Romics Biotechnology. After preprocessing of the raw reads (filtering and QC), the sequencing reads were mapped to the human reference genome version 19 using the Top Hat algorithm version 2.0.9 with Ensemble gene annotations version GRCh37.65. Further analysis was performed with the R statistical programming language version 2.15.0. The DEG-seq package was used to compare differentially expressed transcripts among samples based on FPKM values calculated by Ballgown (fold change≥2.0, Pvalue≤0.05). These down-regulated genes in the shOTUB1 group might be those genes regulated by OTUB1 and ubiquitination degradation signaling pathway, which further were handled with KEGG pathway analysis and GSE function analysis. All up-regulated genes (Table S3) and down-regulated genes (Table S4) in shOTUB1 vs control were presented in the supplementary material.

### T24 Cisplatin-resistance BLCA cell line construction

T24 BLCA cell was cultured with a series of cisplatin concentrations to stimulate the cisplatin resistance, the concentration was designed into the following gradient: 0; 0.5uM; 1.0uM; 3uM; 5uM; 10uM. Finally, we constructed a stable T24 cisplatin-resistance cell by maintaining 3uM cisplatin culture. The related IC50 cell activity experiments were conducted to further identify the reliability and degree of resistance. The constructed T24 cisplatin-resistance cell and control cell were used for subsequent analysis and experiments.

### Animal studies *in vivo*

We transfected EJ cells with OTUB1 OE or β-catenin plasmid or shOTUB1 lentivirus, which further were purified by puromycin screening. Total 2*10^6^ purified cells suspended with 150ul mixture of Matrigel and 1640 culture medium (Matrigel: 1640=1:1) were planted subcutaneously into the nude mice (5-week-old, male Bab1/c mice, Beijing). The tumor size was measured daily for 2 weeks when the tumor diameter reached 2mm. The volume of mice tumors was calculated by the formula 0.52*L*W^2^ (L: the length; W: the width). The subcutaneous tumor-bearing experiments in nude mice were divided into the following groups: control, OTUB1, OTUB1/XAV-939 and β-catenin. At the end of the study, all nude mice were sacrificed and subcutaneous tumors were removed and preserved with formalin. These collected subcutaneous tumors were measured for the comparison of differences in tumor volume between subgroups, these tumors were sliced and performed IHC staining for OTUB1, β-catenin and Ki67 expression levels.

We further transfected constructed cisplatin-resistant T24 BLCA cell line with shOTUB1 lentivirus, further planted subcutaneously control and shOTUB1 cisplatin-resistant T24 BLCA cell line into the nude mice according to the above methods. We successfully constructed a mice model of spontaneous BLCA in our team, KM mice will occur spontaneous BLCA via feeding water supplemented with 0.1% BNN (N-butyl-N-(4-hydroxybutyl) nitrosamine), which has been identified to induce BLCA^20^. These mice were fed BNN for lasting 3mon, stopped feeding BNN and sacrificed mice according to time grouping (group 1: 1-6mon; group 2: 7-12mon). Mice bladders were removed and further handled for H&E staining for organizational structure changes and IHC staining for OTUB1 expression levels. All procedures involving mice were approved by the University Committee on the Use and Care of Animals at Tianjin Medical University and conformed to all regulatory standards.

### Statistical analysis

A t-test was used to compare the differences between the two groups, and P-values less than 0.05 were considered statistically significant for this study. Software GraphPad Prism 8 and ImageJ were used for statistical analysis.

## Results

### OTUB1 is elevated in BLCA and related to poor prognosis

**Figure 1:**
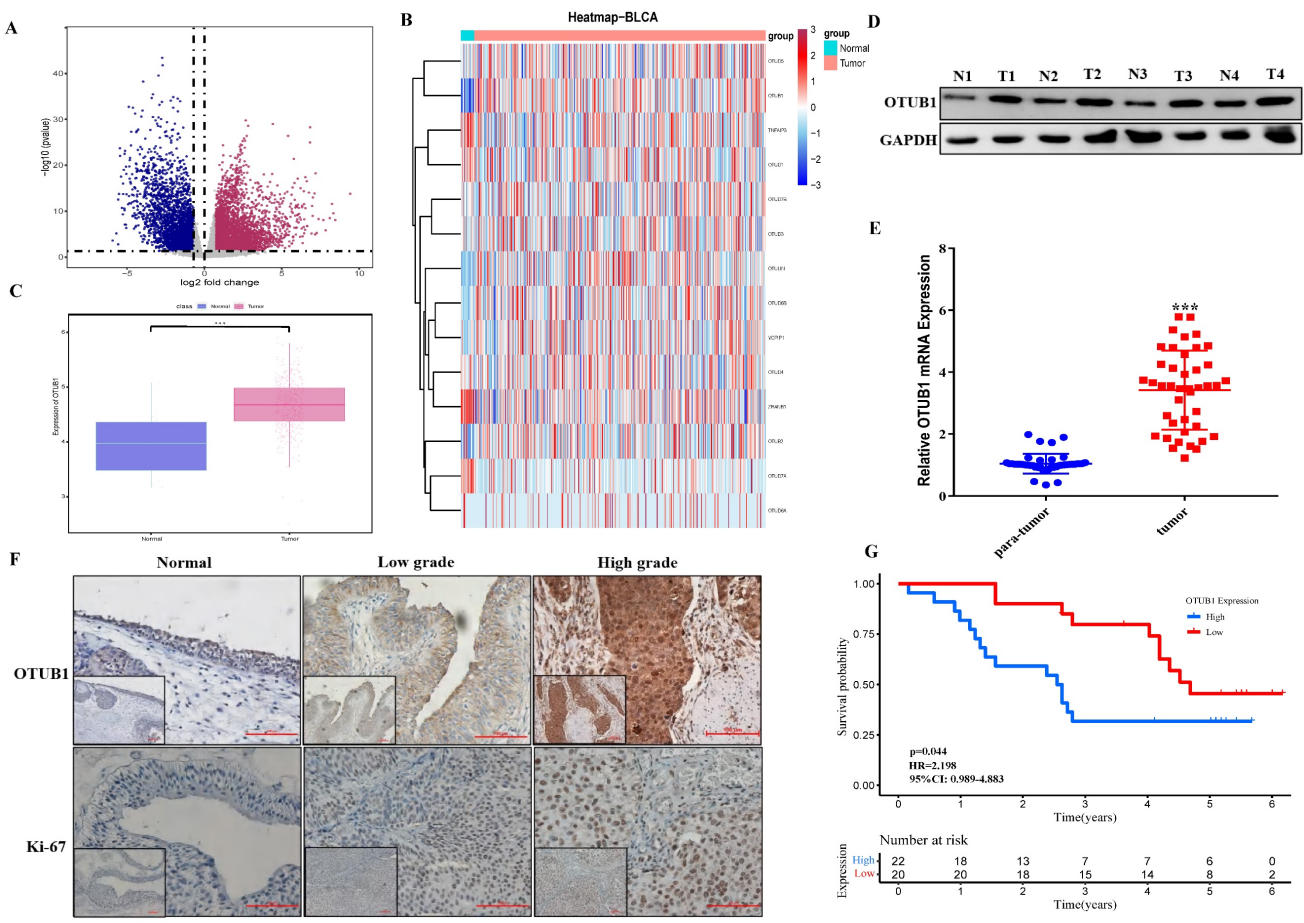
OTUB1 is elevated in BLCA and related to poor prognosis. A. Volcano plot of differentially expressed genes (DEGs) in TCGA BLCA data. B. The heatmap showed the expression of all members in the OTU deubiquitinase superfamily in BLCA and para-cancer tissues. C. Box plots of the relative mRNA expression levels of OTUB1 in BLCA and para-cancer samples from the TCGA database. D, E. The analysis of OTUB1 protein and mRNA expression in BLCA (T) and paired normal bladder (N) tissues by western blot and qRT- PCR. F. Representative immunohistochemical staining of OTUB1 and ki-67 in BLCA and paired normal bladder tissues (100X, 200X). G. Kaplan-Meier plots representing survival probabilities in 42 BLCA patients according to the relative expression level of OTUB1. Statistical analysis was conducted using the Student t-test and log-rank test.

The fact that OTUB1 functions critical tumor regulator have been known recently, while the relationship between OTUB1 and BLCA development remains unclear. In this study, we systematically analyzed all gene expression profiles in The Cancer Genome Atlas (TCGA) BLCA database and found that multiple gene expression was changed significantly in BLCA tissue compared with normal bladder tissue (Fig. 1A; Table S1-2). Further analysis showed that most members' expression of the OTU deubiquitinase family was dysregulated in BLCA tissue, OTUB1 was the most prominent among all OTU deubiquitinase members because of its significantly elevated levels in BLCA tissue compared with lower expression in normal bladder tissue (Fig. 1B, C, Fig. S1). To further verify TCGA results, we collected 42 paired BLCA tissue and para-cancer tissue from the department of urology in our hospital. Total protein and RNA, extracted from these samples, were applied for western blot and RT-PCR assay for OTUB1 expression detection in BLCA tissue and para-cancer tissue. The results showed that OTUB1 expression was elevated significantly in BLCA tissue compared with the normal tissue (Fig. 1D, E), which was consistent with the database. The results of OTUB1 immunohistochemical staining in BLCA tissue and normal bladder tissue from the online database and our collected samples showed that OTUB1 was up-regulated in BLCA tissue compared to the normal bladder tissue, this elevated expressed phenomenon was also established in diagnosed low- and high-grade-bladder-cancer (Fig. 1F, Fig. S2A). To further explore the association between OTUB1 expression in BLCA and clinical significance, we collected and analyzed all 42 BLCA patient information, the results showed that elevated OTUB1 expression was significantly related to tumor size and poorer prognosis (Fig. 1G, Table S5). These results implied that OTUB1 was elevated in BLCA tissue and associated with a poor prognosis.

### Elevated OTUB1 is involved in tumorigenesis of BLCA *in vivo*

**Figure 2:**
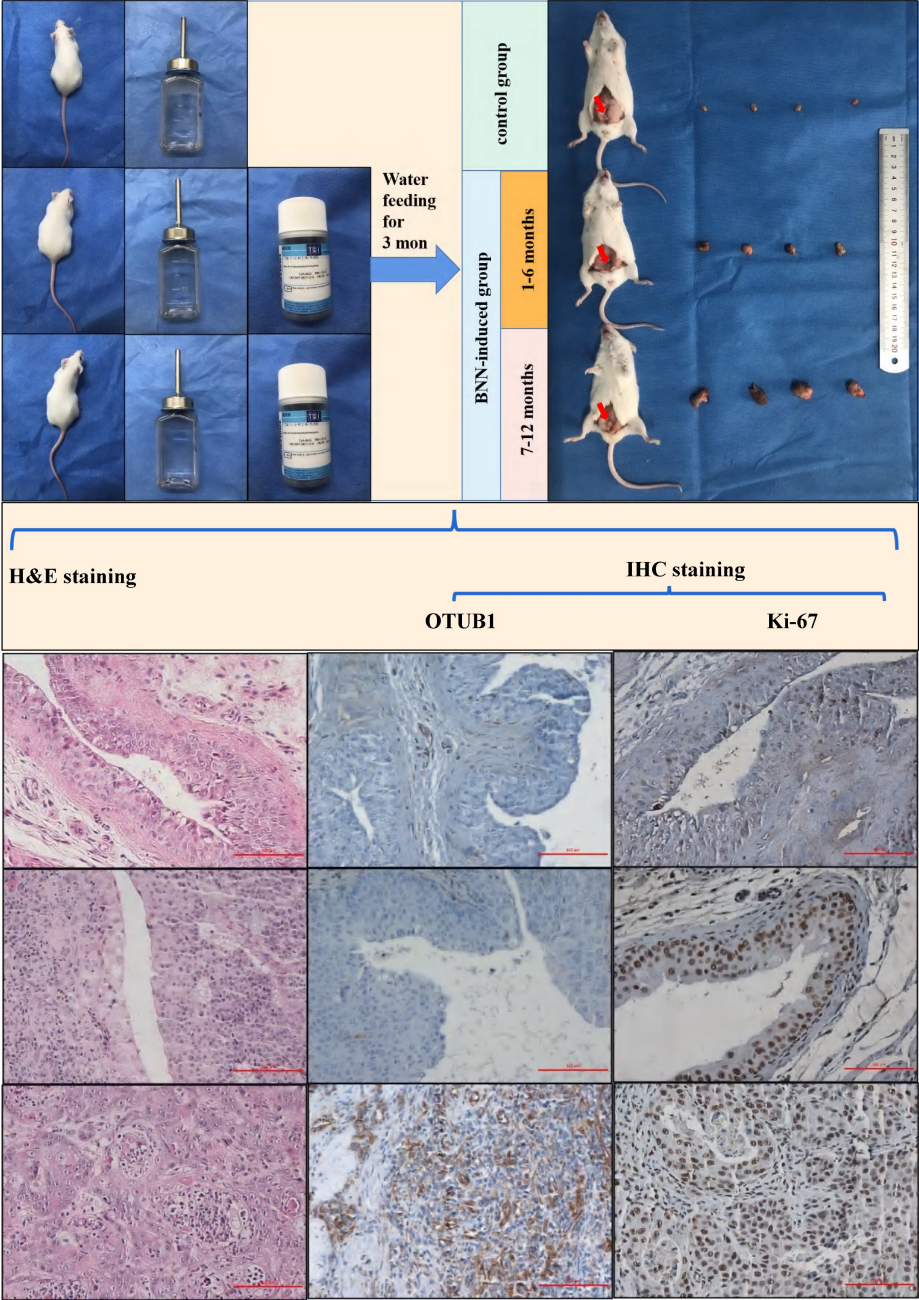
Elevated OTUB1 is involved in the tumorigenesis of BLCA *in vivo*. Flow chart for the mouse model of spontaneous BLCA by feeding 0.1% BNN in water, these mice were treated with or without 0.1% BNN daily for 3 months. After stopping feeding BNN, all mice were divided into two groups according to time (1-6 months group and 7-12 months group). These bladders were removed and further handled for H&E staining and immunohistochemical staining (OTUB1 and ki-67). The left group from top to bottom: normal bladder structure, bladder carcinoma *in situ*, invasive BLCA; the Middle group from top to bottom: IHC staining for OTUB1 according to the sequence of H&E staining; Right group from top to bottom: IHC staining for ki-67 according to the sequence of H&E staining.

Mice BLCA was induced via feeding 0.1% BNN (N-butyl-N-(4-hydroxybutyl) nitrosamine) in water, these mice were fed in water with or without BNN for three months. After 3 months, these mice were sacrificed according to different time groups (group 1: stopping feeding 1-6 months; group 2: stopping feeding 7-12 months), removed bladder tissues and saved in formalin for subsequent analysis. These mice's bladder tissue was further embedded with paraffin and sliced for H&E staining and immunohistochemical staining. The results showed that the bladder volume of the BNN-induced group was significantly bigger compared to that in the control group, and the bladder volume in the 7-12 months group also was bigger than that in the 1-6 months group (Fig. 2). The H&E staining results (H&E staining image from top to bottom) demonstrated that the normal bladder structure without abnormal proliferation or pathological changes in the control group have been observed, while the pathological changes of bladder carcinoma *in situ* were found in 1-6 months group; and the pathological changes of invasive high-grade BLCA were observed in 7-12 months group (Fig. 2). Based on the above results, the BNN-induced BLCA model was proved to be successful and effective, and the severity of BLCA may be affected by the time effect. The results were further consolidated by immunohistochemical staining in proliferation-related marker ki-67, we found that ki-67 expression increased gradually in mice bladders from the control group, 1-6 months group to 7-12 months group (Fig. 2; ki-67 IHC image from top to bottom). Interestingly, we found that OTUB1 expression was significantly higher in the BNN-induced group mice bladder compared with that in the control group mice bladder, especially in invasive BLCA of 7-12 months group (Fig. 2; OTUB1 IHC image from top to bottom). All the above results implied that OTUB1 might be involved in the tumorigenesis of BLCA, elevated OTUB1 expression in bladder tissue might be the potential driving factor to BLCA tumorigenesis and progression, and targeting OTUB1 might be a potent strategy to intervene or inhibit BLCA occurrence and development. The detailed role and mechanism of OTUB1 still need more effort and research.

### OTUB1 facilitates the proliferation and migration of BLCA

**Figure 3:**
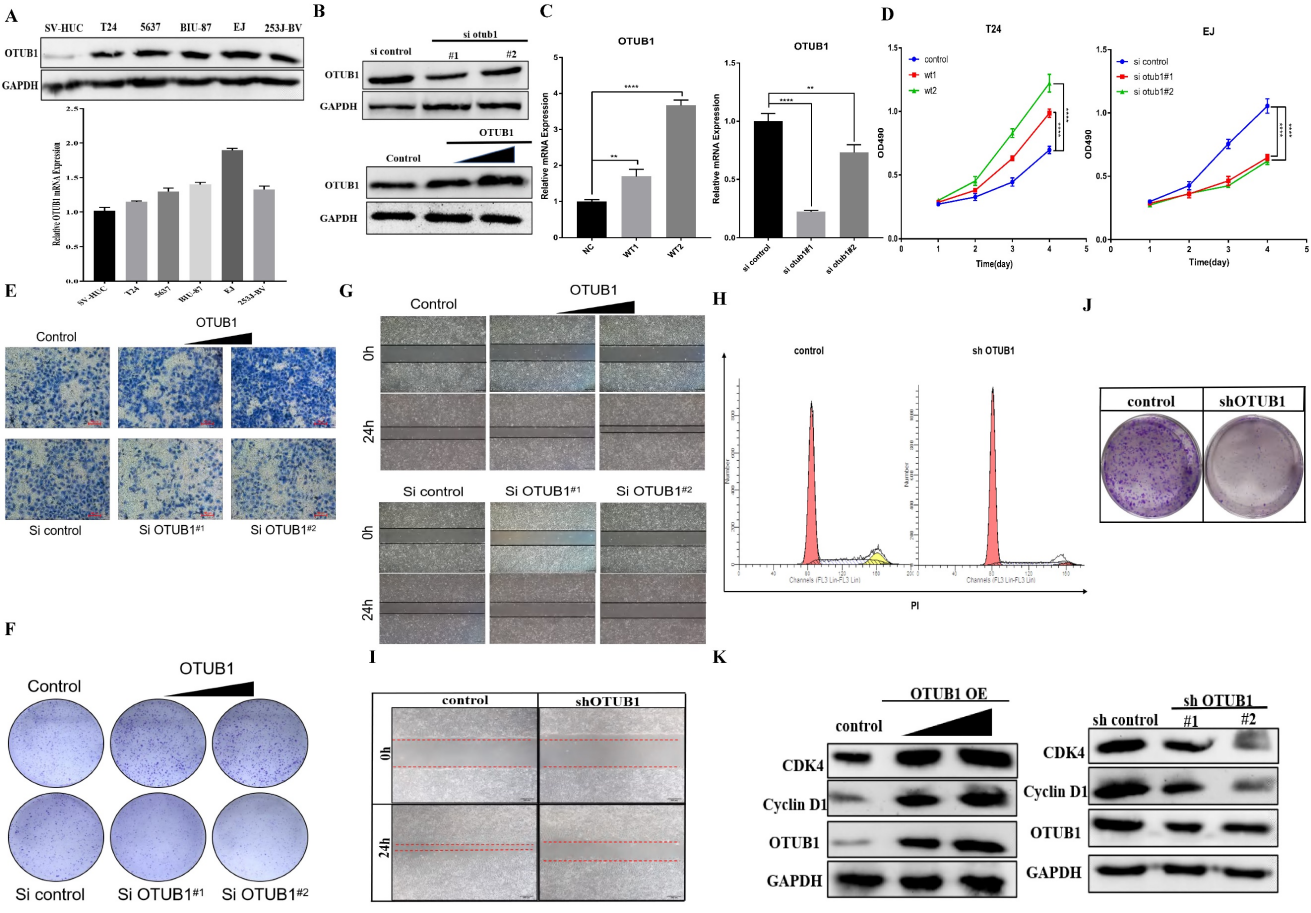
OTUB1 facilitates the proliferation and migration of BLCA. A. Relative OTUB1 expression in bladder epithelial immortalized cell SV-HUC and several BLCA cells by western blot and qRT-PCR. B, C. Relative OTUB1 expression following overexpressed or knockdown OTUB1 by western blot. D, F, G. MTT assay, clone formation experiments and healing assay showed the proliferation ability of BLCA cells following overexpressed or knockdown OTUB1. E. Transwell assay showed the invasion ability of BLCA cells following overexpressed or knockdown OTUB1. H, I, J. Cell cycle assay, clone formation experiments and healing assay showed the proliferation ability of BLCA cell after transfecting with shOTUB1 lentivirus. K. Relative cell cycle-related markers expression following overexpressed or knockdown OTUB1 by western blot.

To further explore OTUB1 effects on the proliferation, migration and progression in BLCA, a series of phenotype-related experiments were conducted. We found that OTUB1 protein and RNA expression in BLCA cells (including T24, 5637, BIU-87, EJ and 253J-BV) was higher than in human bladder epithelial immortalized cell (SV-HUC) (Fig. 3A), which was confirmed the clinical sample expressed information. We transfected T24 and EJ cells with gradient OTUB1 OE plasmid and OTUB1 siRNA respectively, OTUB1 expression levels were induced gradually by OTUB1 OE plasmid and restrained by designed siRNA (Fig. 3B, C). The transfected T24 and EJ cells were used for further cancer phenotype experiments, including MTT assay, transwell assay, colony forming assay and wound healing assay. The results of the MTT assay showed that elevated OTUB1 promotes significantly the proliferation ability, while decreased OTUB1 plays the opposite role (Fig. 3D). We additionally discovered that OTUB1 enhances the migratory capacity of BLCA cells, as evidenced by the Transwell results obtained with T24 cells (Fig. 3E). This observation was further supported by subsequent colony-forming assays (Fig. 3F) and wound healing assays (Fig. 3G), which reaffirmed OTUB1's role in promoting the proliferation of BLCA cells. To further verify this hypothesis, we continued to transfect the BLCA EJ cell with the designed shOTUB1 lentivirus, cell was screened and purified by puromycin. Cell cycle results showed that decreased OTUB1 regulates proliferation and cell cycle distribution, which prolonged the G1 phase percentage and shortened the G2/S phase ratio (Fig. 3H). The results implied that OTUB1 regulates BLCA proliferation ability via mediating cell cycle development. Related phenotype experiments also proved that OTUB1 promotes significantly the proliferation and progression of BLCA, which was consistent with previous results (Fig. 3I, J). In addition, elevated OTUB1 enhanced the expression of cell cycle-related factors, such as cyclin D1 and cyclin-dependent kinase 4, and decreased OTUB1 represented the opposed effects (Fig. 3K).

### OTUB1 interacts with β-catenin and regulates its stability

To deeper understanding of the biological and clinical significance of OTUB1-mediated deubiquitination on the occurrence and progression in BLCA, we analyzed the transcript and gene expression profile of control vs shOTUB1 EJ cell by high-throughput RNA sequencing (RNA-seq).

**Figure 4:**
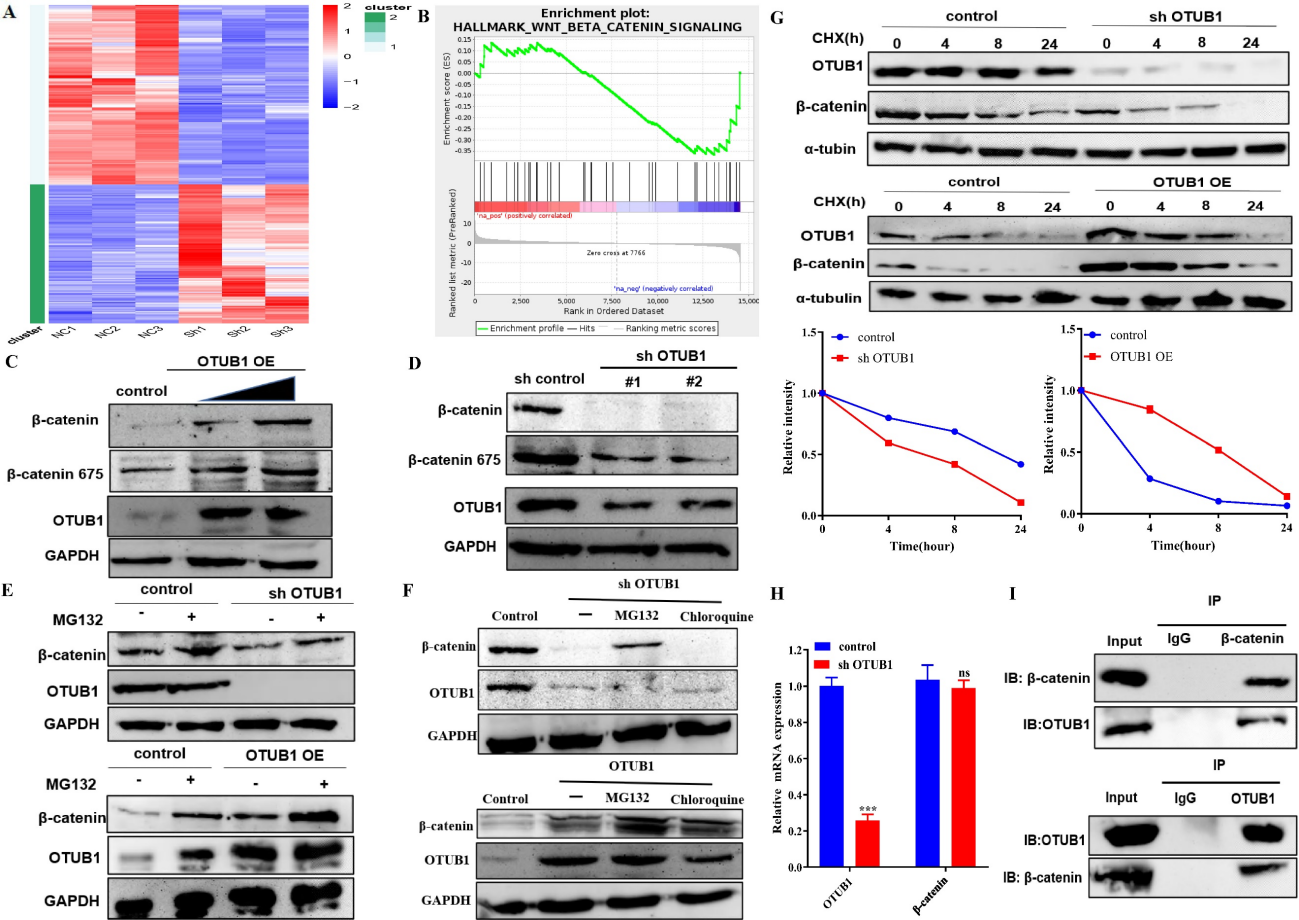
OTUB1 interacts with β-catenin and regulates its stability. A. Heatmap of RNA-seq expression data for EJ cell transfected with control or shOTUB1 lentivirus. B. GSEA of RNA-seq data revealed that OTUB1 target genes were involved in Wnt/β-catenin signaling pathway. C, D. Relative expression of β-catenin following overexpressed or knockdown OTUB1. E. Relative expression of β-catenin following overexpressed or knockdown OTUB1 (treated with or without MG132 for 8h). F. Relative expression of β-catenin following overexpressed or knockdown OTUB1 (treated with MG132 or chloroquine for 8h). G. Relative expression of β-catenin following overexpressed or knockdown OTUB1 (treated with or without CHX for 0h, 4h, 8h and 24h). H. Relative mRNA expression of OTUB1 and β-catenin following knockdown OTUB1. I. The interaction between OTUB1 and β-catenin was determined by a co-immunoprecipitation assay.

Protein and RNA expression of OTUB1 were reduced significantly in the shOTUB1 group compared with the control group (Fig. S3A), top-100 up- and down-regulated genes were presented in the heatmap (Fig. 4A), all up-regulated 151 genes (Table S3) and down-regulated 196 genes (Table S4) were listed in the supplementary material. Gene Ontology (GO) analysis showed that enriched genes were mainly involved in cell adhesion and multiple intracellular biological processes (Fig. S3C), implying that OTUB1 regulates multiple biological and pathological processes, which further promotes the crucial role of OTUB1. Kyoto Encyclopedia of Genes and Genomes (KEGG) pathway analysis revealed that OTUB1 is involved in various important signaling pathways, such as transcriptional regulation, PI3K-AKT signaling pathway, cancer-related pathway, TGF-β signaling pathway, TNF pathway, Hippo signaling pathway and so on (Fig. S3D). Further experiments and analysis showed that these down-regulated genes in the shOTUB1 group might be regulated by ubiquitination degradation and OTUB1-mediated deubiquitination, which are the main objects of subsequent analysis. Gene set enrichment analysis (GSEA) was used to search OTUB1-mediated signaling pathways in BLCA, including the wnt/β-catenin signaling pathway (Fig. 4B) and epithelial-mesenchymal transition signaling pathway (Fig. S3E). The wnt/β-catenin signaling pathway is the classic and famous pathway, which regulates various pathophysiological processes and multiple genes transcription regulation. The relationship between OTUB1 and the wnt/β-catenin signaling pathway remains unclear, previous studies indicated that OTUB1 binds with β-catenin and regulates its degradation in colorectal cancer. In this study, we further explore a deeper association between OTUB1 and β-catenin, and the role of the OTUB1/β-catenin axis on carcinogenesis and progression of BLCA, which will be a more complete supplement for OTUB1 complicated functions. To further explore the specific relationship between OTUB1 and β-catenin, the network association involved in OTUB1 and β-catenin was predicted via GENEMANIA online database, and the results showed that molecular and functional interaction between OTUB1 and β-catenin was undoubtedly (Fig. S3B). Further correlation analysis also consolidated the conclusion, that OTUB1 expression was positively related to β-catenin expression (Fig. S3C, R=0.18, P=0.00037). To further verify the results of the database, we transfected T24 and EJ cells with gradient OTUB1 plasmid and shOTUB1 lentivirus respectively, and the expression of β-catenin was induced or inhibited correspondingly (Fig. 4C, D). The results implied that β-catenin expression was regulated by OTUB1, the regulatory manner needs further verification. Due to the unique deubiquitinase activity of OTUB1, we transfected T24 and EJ cells with gradient OTUB1 plasmid and shOTUB1 lentivirus respectively coupled with 8h 10uM protease inhibitor (MG132) treatment. The results indicated that β-catenin expression was decreased significantly in the shOTUB1 group compared with the control group, while MG132 partially preserve its expression, indicating that OTUB1 influenced the expression of β-catenin in a proteasome-dependent manner; OTUB1 OE group indicated the opposed effects (Fig. 4E). To further exclude the lysosomal protein degradation pathway, we continued to treat shOTUB1 cells with 10uM MG132 and 200uM chloroquine (a specific lysosomal enzyme inhibitor) for 8 h. We found that β-catenin expression decreased significantly, MG132 could partially recover its expression, while chloroquine could not (Fig. 4F). To further verify the hypothesis that OTUB1 regulates β-catenin protein ubiquitination level, we treated T24 and EJ cells transfected OTUB1 plasmid and shOTUB1 lentivirus respectively with 10mg/ml cycloheximide (protein synthesis inhibitor) at 0h, 4h, 8h and 24h. The results showed that β-catenin expression decreased more significantly in the shOTUB1 group compared with that in the control group, the OTUB1 OE group presented the opposed effects (Fig. 4G). To exclude the effects of transcriptional regulation, we conducted RT-PCR and found that decreased OTUB1 failed to inhibit β-catenin mRNA expression (Fig. 4H). The above results implied that OTUB1 promotes β-catenin expression and stability at the protein level instead of the mRNA level. A co-immunoprecipitation experiment was performed to further determine the specific relationship between OTUB1 and β-catenin, and the results showed that OTUB1 binds with β-catenin (Fig. 4I). To further determine the correlation between OTUB1 and β-catenin, the expression of OTUB1 and β-catenin in BLCA and normal bladder tissue were found to be consistent in The Human Protein Atlas (Fig. S2A). We further collected BLCA pathological specimens and para-cancer tissue for immunohistochemical staining of OTUB1 and β-catenin in our hospital, the results showed that the expression of OTUB1 and β-catenin was consistent (Fig. S2B-D), OTUB1 was closely related to β-catenin (Fig. S2E).

### OTUB1 maintains β-catenin stability depending on its deubiquitinase activity

**Figure 5:**
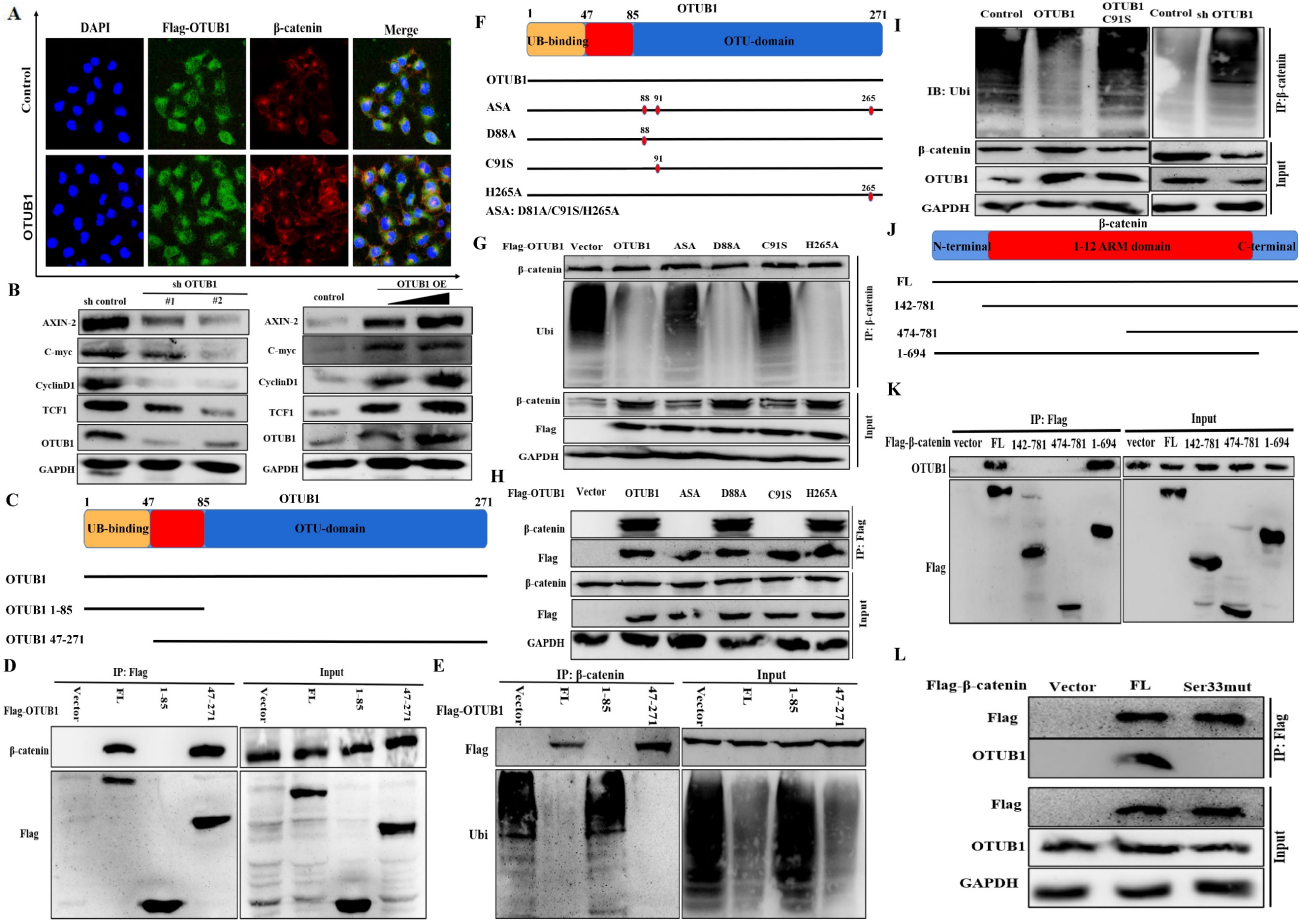
OTUB1 maintains β-catenin stability depending on its deubiquitinase activity. A. Immunofluorescence assay showed the nuclear expression of β-catenin in BLCA cells transfected with OTUB1 plasmid. B. Relative expression of Wnt/β-catenin downstream targets following overexpressed or knockdown OTUB1. C. Schematic showed the functional domain and two mutant fragments in OTUB1. D, E. Immunoprecipitation assay showed that the 47-271 domain in OTUB1 interacts with β-catenin, further deubiquitinates and stabilizes β-catenin. F. Schematic showed the three amino acid mutant sites in OTUB1 (D88A; C91S; H265A; ASA: D88A, C91S and H265A). G, H. Immunoprecipitation assay showed that D88A and H265A in OTUB1 interact with β-catenin, further deubiquitinates and stabilize β-catenin; while C91S and ASA in OTUB1 do not. I. Immunoprecipitation assay showed that overexpressed OTUB1 restrains the ubiquitination of β-catenin, knockdown OTUB1 promotes the ubiquitination of β-catenin, while OTUB1 C91S has no effects. J. Schematic showed the functional domain and three mutant fragments in β-catenin. K. Immunoprecipitation assay showed that 1-694 domain fragment in β-catenin interacts with OTUB1. L. Immunoprecipitation assay showed that β-catenin serine33 mutation fails to interact with OTUB1.

The fact that OTUB1 binds with β-catenin has been identified, and the results of immunofluorescence showed that elevated OTUB1 promotes β-catenin expression and its nuclear import, which further regulates the complicated transcription processes (Fig. 5A). To further explore the effects of OTUB1 on the Wnt/β-catenin signaling pathway, several downstream proteins (including AXIN-2, C-myc, cyclin D1 and TCF1) were found to decrease in the shOTUB1 group compared with the control group, while overexpressed OTUB1 presented the opposed effects (Fig. 5B). Previous studies have proved that OTUB1 restrains target protein ubiquitination through two independent manners: one is to remove directly the ubiquitin chain from substrate protein depending on its deubiquitinase catalytic activity; another is to restrain the transfer from E2 ubiquitin-conjugating enzyme to E3 ubiquitin ligase depend on the ligation activity of N-terminal in OTUB1 to E2 ubiquitin-conjugating enzyme. Based on the above observations, we sought to identify which OTUB1 regions are critically required for its interaction with β-catenin. We generated two OTUB1 domain fragments (Fig. 5C). The C-terminal OTU domain of OTUB1 was identified to mediate its association with β-catenin via Co-IP assays (Fig. 5D, E). The results demonstrated that OTUB1 binds with β-catenin via the canonical manner depending on the deubiquitinase catalytic activity. We continued to construct the mutation of three critical amino acid sites (including D88A, C91S and H265A) in OTUB1 and found that C91S mutation cannot restrain the ubiquitination of β-catenin (both C91S and ASA) (Fig. 5F-H). The results further demonstrated that the C91 amino acid site plays a core role in the interaction between OTUB1 and β-catenin. The results further showed that OTUB1 restrains significantly the ubiquitination of β-catenin; while OTUB1 C91S cannot; decreased OTUB1 promotes the ubiquitination level of β-catenin (Fig. 5I). To explore the binding site of β-catenin to OTUB1, we constructed several domain fragments and found 1-694 aa fragment binds with OTUB1, while others cannot (Fig. 5J, K). The phosphorylation of serine^33^ of β-catenin has been known to be involved in the degradation, we further found that serine^33^ mutation cannot bind with OTUB1 in subsequent experiments (Fig. 5L)^.^ Based above results, we identified that OTUB1 (via 47-271 aa) binds with β-catenin (via 1-694 aa) and restrains its ubiquitination degradation; C91 in OTUB1 and Ser33 in β-catenin were the critical sites for the interaction and physiological function.

### OTUB1 promotes the progression of BLCA depending on β-catenin/RIPK3/MLKL/necroptosis signaling pathway

**Figure 6:**
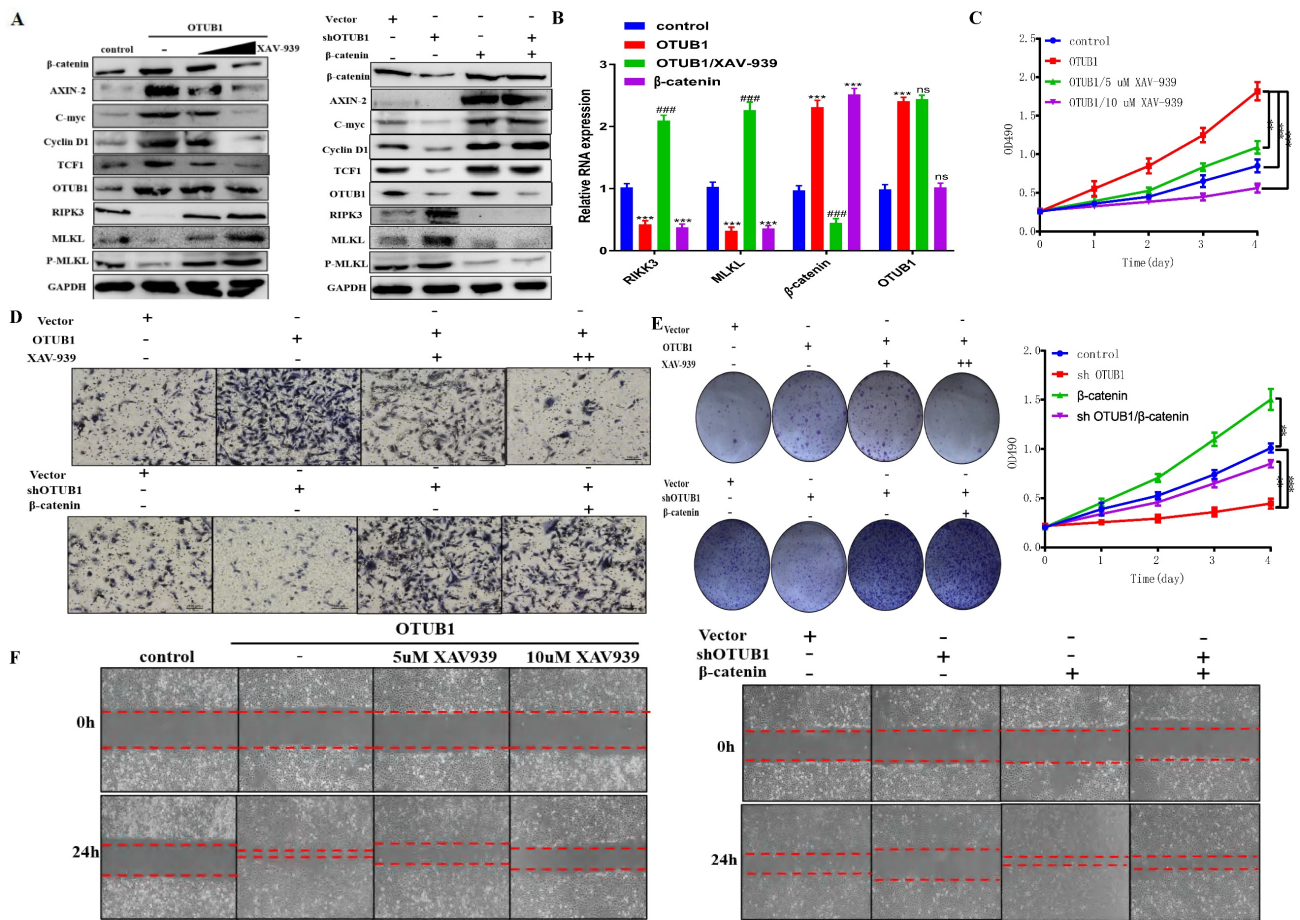
OTUB1 promotes the progression of BLCA depending on β-catenin/RIPK3/MLKL/necroptosis signaling pathway. A. Relative expression of OTUB1, β-catenin and downstream targets following overexpressed OTUB1 with or without XAV-939 (5uM, 10uM) treatment; relative expression of OTUB1, β-catenin and downstream targets following knockdown OTUB1 and/or overexpressed β-catenin. B. The RNA expression of OTUB1, β-catenin, RIPK3 and MLKL in elevated OTUB1, β-catenin groups with or without XAV-939 treatment. C, E, F. MTT assay, clone formation experiments and healing assay showed the proliferation ability of BLCA cell following overexpressed OTUB1 with or without XAV-939 (5uM, 10uM) treatment; coupled with that following knockdown OTUB1 and/or overexpressed β-catenin. D. Transwell assay showed the invasion ability of BLCA cells following overexpressed OTUB1 with or without XAV-939 (5uM, 10uM) treatment; coupled with that following knockdown OTUB1 and/or overexpressed β-catenin.

To further verify the regulatory relationship between OTUB1 and β-catenin in BLCA, we introduced XAV-939 (a β-catenin inhibitor) to subsequent experiments. The results showed that the expression of β-catenin and its downstream targets (AXIN-2, C-myc, cyclin D1 and TCF1) were induced by OTUB1 overexpression, which was further restrained as the concentration of XAV-939 gradually increased, OTUB1 expression has no significant changes (Fig. 6A). On other hand, the expression of β-catenin and its downstream targets were restrained by shOTUB1, which was rescued by elevated β-catenin (Fig. 6A). Moreover, previous studies have proved that necroptosis is associated with tumorigenesis and progression in multiple cancers. The expression of typical markers (such as RIPK1, RIPK3 and MLKL) and their phosphorylation are the critical nodes of necroptosis. To further explore the relationship between OTUB1/β-catenin complex with necroptosis, the results showed that OTUB1/β-catenin restrained significantly the expression of RIPK3, MLKL and P-MLKL, while XAV-939 rescued this phenomenon (Fig. 6A). These results implied that OTUB1 promotes the progression of BLCA via mediating the β-catenin/necroptosis signaling pathway. To verify this hypothesis, the results further showed that the RNA expression of RIPK3/MLKL was inhibited by increased OTUB1 and β-catenin, while induced by XAV-939 (Fig. 6B). The conclusion that necroptosis-related markers expression was restrained by the OTUB1/β-catenin axis at the transcriptional level has been preliminary confirmed. The results showed the line regulatory network that OTUB1 stabilizes β-catenin, while β-catenin cannot regulate OTUB1 expression. Further results demonstrated that OTUB1 facilitates the proliferation and invasion of BLCA through stabilizing β-catenin expression, which was restrained by decreased β-catenin or XAV-939 (Fig. 6C-F). The results implied that the OTUB1/β-catenin axis promotes the tumorigenesis and progression of BLCA, targeting the OTUB1/β-catenin axis or applying XAV-939 might be the potential therapeutic strategy for BLCA patients.

### OTUB1 is involved in cisplatin resistance of BLCA through stabilizing β-catenin

**Figure 7:**
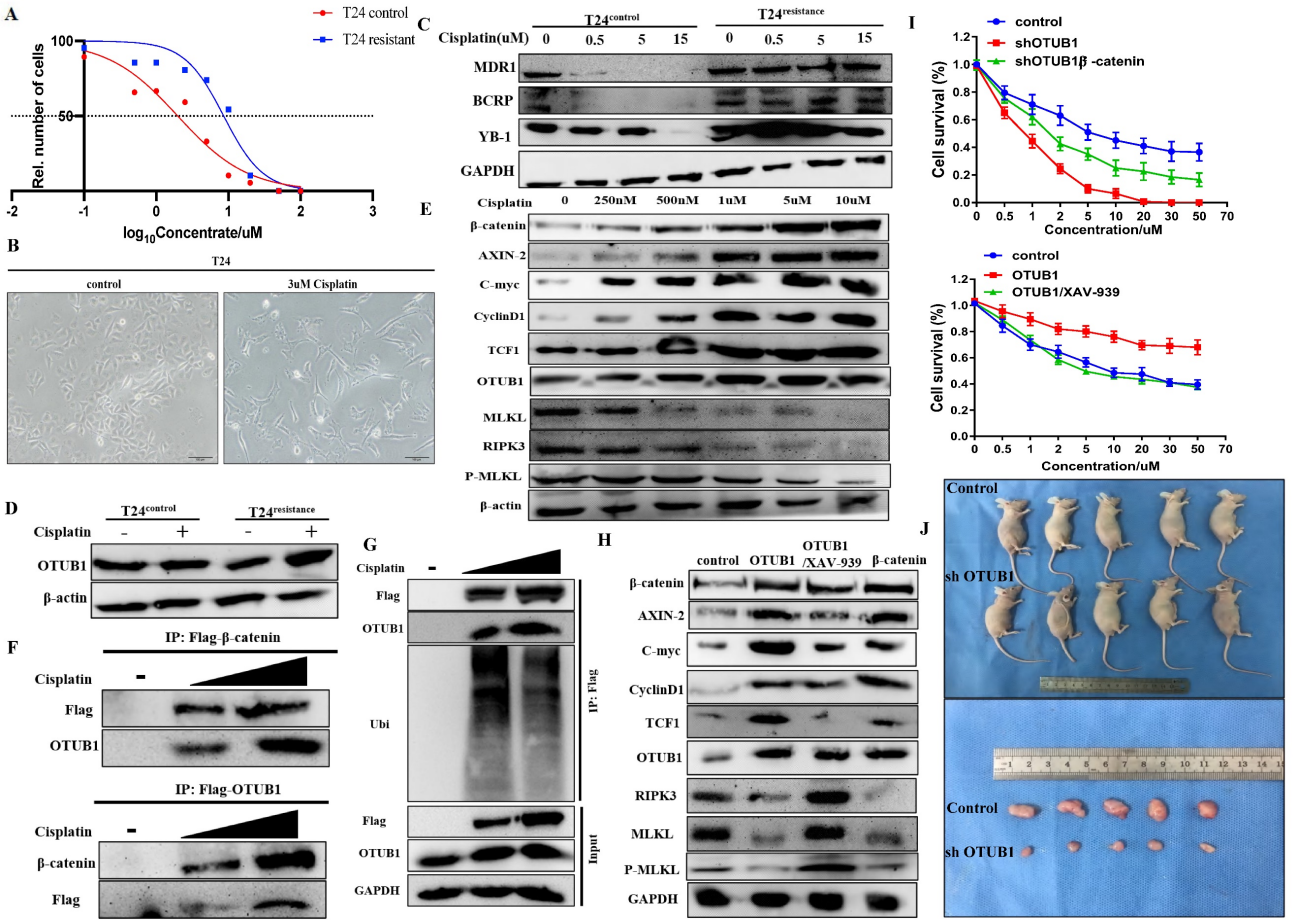
OTUB1 is involved in cisplatin resistance of BLCA through β-catenin stabilization. A. Cell survival assay was determined in T24 control cell and T24 cisplatin-resistance cell. B. The changes in cell morphology between the T24 control cell and the T24 cisplatin-resistance cell. C. Relative expression of several chemoresistance markers (including MDR1, BCRP and YB-1) in T24 control cell and T24 cisplatin-resistance cell with or without cisplatin treatment. D. Relative expression of OTUB1 in T24 control cell and T24 cisplatin-resistance cell with or without cisplatin treatment. E. Relative expression of OTUB1, necroptosis-related markers (such as RIPK3, MLKL and P-MLKL), β-catenin and downstream proteins (including AXIN-2, C-myc, cyclin D1 and TCF1) in T24 cisplatin-resistance cells treated with gradient cisplatin concentration. F. Immunoprecipitation assay showed the relationship between OTUB1 and β-catenin in T24 cisplatin-resistance cells. G. Immunoprecipitation assay showed that gradient cisplatin concentration promotes the interaction between OTUB1 and β-catenin, and restrains the ubiquitination of β-catenin. H. Relative expression of OTUB1, necroptosis-related markers (such as RIPK3, MLKL and P-MLKL), β-catenin and downstream targets following elevated OTUB1, β-catenin with or without XAV-939 treatment in T24 cisplatin-resistance cell (control, OTUB1, OTUB1/XAV-939, β-catenin). I. Cell survival assay was determined in T24 cisplatin-resistance cells following overexpressed OTUB1 with or without 10uM XAV-939 (or knockdown OTUB1 with or without overexpressed β-catenin). J. Knockdown OTUB1 restrains the growth in T24 cisplatin-resistance mice bladder tumor *in vivo*.

The fact that the canonical Wnt/β-catenin signaling pathway is involved in the chemoresistance of several cancers has been identified. Thus, we next explored whether OTUB1 influences the therapeutic response to chemotherapy in BLCA. Cisplatin is the most common chemotherapy drug for BLCA, we constructed T24 cisplatin-resistance BLCA cells (T24^resistance^) for subsequent analysis (Fig. 7A, B). To further verify the chemoresistance-reliability of the T24^resistance^ cell, several common chemoresistance markers (including MDR1, BCRP and YB-1) were selected for analysis. The results showed that the expression of MDR1, BCRP and YB-1 gradually declined in T24^control^ cells with cisplatin concentration increased, while T24^resistance^ cells did not (Fig. 7C). Compared with the T24^control^ group, OTUB1 expression was induced by cisplatin in the T24^resistance^ group (Fig. 7D). To further determine the role of the OTUB1/β-catenin axis in cisplatin resistance in BLCA, we treated the T24^resistance^ cell with the increasing-concentration cisplatin, the results showed that the expression of OTUB1, β-catenin and downstream targets were gradually increased by gradient cisplatin concentration in T24^resistance^ cell, while the results of RIPK3 and MLKL were the opposite (Fig. 7E). The results were consolidated with the previous results, which further support the hypothesis that OTUB1 promotes the development of BLCA by mediating the β-catenin/RIPK3/MLKL signaling pathway. To explore the mechanism of the OTUB1/β-catenin axis in T24^resistance^ cells, the immunoprecipitation assay between OTUB1 and β-catenin was conducted, and the results further confirmed the hypothesis that OTUB1 binds with β-catenin to facilitate BLCA progression and cisplatin resistance (Fig. 7F). Subsequent experiments further identified that OTUB1 deubiquitinated and stabilized β-catenin in the T24^resistance^ cell (Fig. 7G). To further explore the function of the OTUB1/β-catenin axis in the cisplatin-resistance occurrence, we transfected the T24^resistance^ cell with OTUB1, β-catenin, or treated the T24^resistance^ cell with XAV-939. The results showed that the expression of β-catenin and its targets was increased by elevated OTUB1 and β-catenin, while increased expression was further restrained by XAV-939 in the T24^resistance^ cell (Fig. 7H). Consistent with the previous results, the expression of RIPK3, MLKL and P-MLKL was restrained by elevated OTUB1 and β-catenin, while induced by XAV-939 (Fig. 7H). The results of the cell survival assay showed that decreased OTUB1 attenuates the chemoresistance ability in the T24^resistance^ cell, which is rescued by elevated β-catenin (Fig. 7I). On other hand, elevated OTUB1 facilitates the chemoresistance in the T24^resistance^ cell, which is conversely restrained by XAV-939 (Fig. 7I). Based on the above results, the fact that OTUB1 promotes cisplatin resistance in BLCA through mediating β-catenin has been confirmed. Furthermore, we planted T24^resistance^ cells transfected with shOTUB1 in nude mice subcutaneously and found that the volume of tumors in the shOTUB1 group is significantly restrained compared with that in the control group (Fig. 7J). Taken together, these findings support the important role of OTUB1 in regulating the response of cancer cells to cisplatin chemotherapy through β-catenin stabilization.

### Targeting the OTUB1/β-catenin/RIPK3/MLKL axis suppresses the tumorigenesis and progression of BLCA *in vivo*

**Figure 8:**
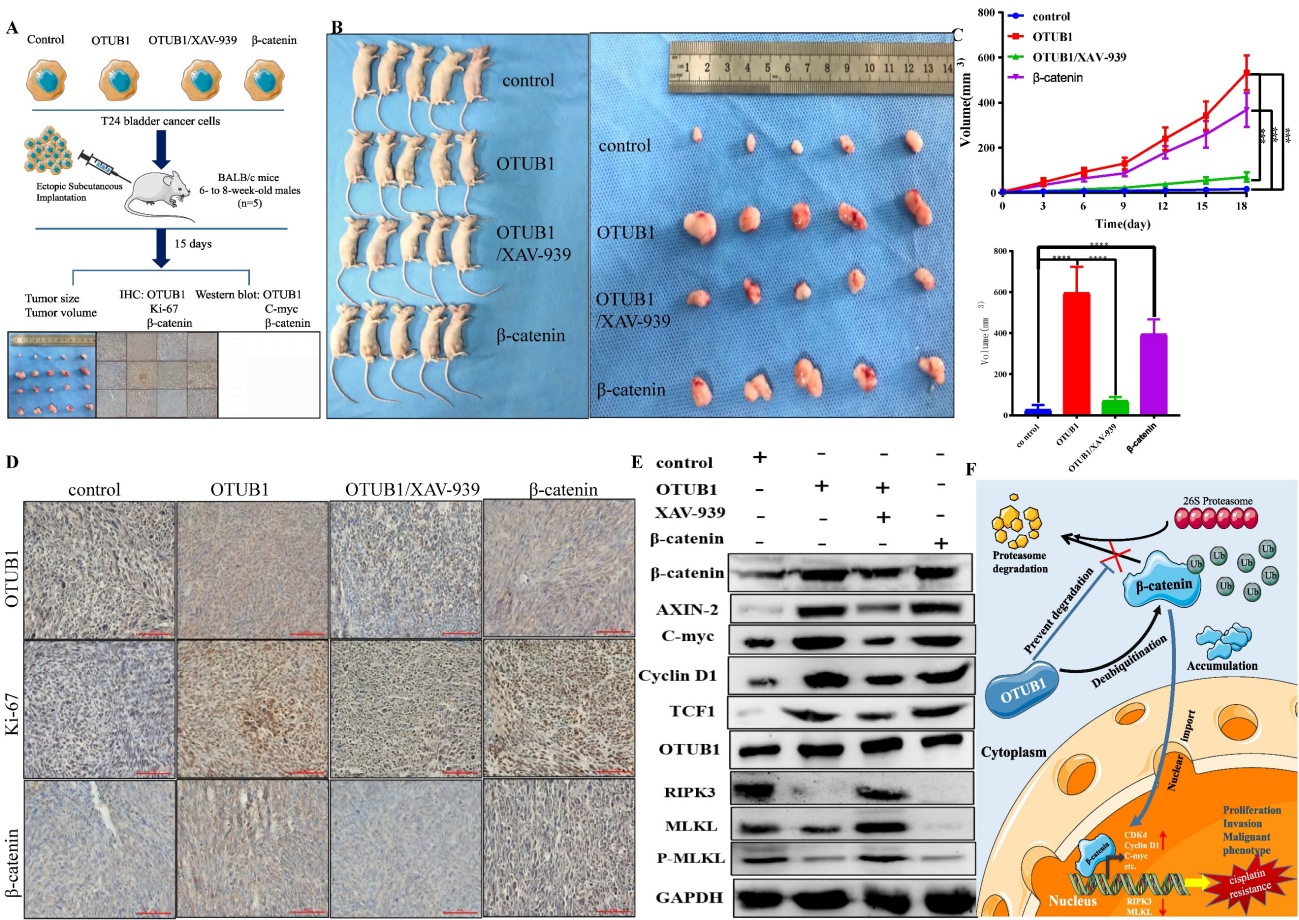
Targeting the OTUB1/β-catenin axis suppresses the tumorigenesis and progression of BLCA *in vivo*. A. The flow chart to experimental design for the *in vivo* tumorigenesis assay in four different bladder cell types (control, OTUB1, OTUB1/XAV-939, β-catenin). B, C. The volume and growth of tumors in the following groups (control, OTUB1, OTUB1/XAV-939, β-catenin). D. Immunohistochemical staining showed the expression of OTUB1, ki-67 and β-catenin in mice tumors among control, OTUB1, OTUB1/XAV-939 and β-catenin groups. E. Relative expression of OTUB1, necroptosis-related markers (such as RIPK3, MLKL and P-MLKL), β-catenin and downstream targets in mice tumors among control, OTUB1, OTUB1/XAV-939 and β-catenin groups. F. Simplified schematic to a working model for regulation of β-catenin stability and cancer tumorigenesis and chemoresistance by OTUB1.

To further consolidate the core role of the OTUB1/β-catenin axis in the tumorigenesis and chemoresistance of BLCA, we next implanted T24 BLCA cell transfected designed plasmid in subcutaneous nude mice. The implanted cells are divided into the following groups: control group; elevated OTUB1 group; elevated OTUB1 coupled with XAV-939 treated group; β-catenin overexpression group. When the diameter of the tumor reaches 2mm, the volume of the tumor begins to be measured every three days for 15 days (Fig. 8A). At the end of time, all mice were sacrificed and tumors were removed, these tumors were further processed for subsequent western blot and immunohistochemical staining (Fig. 8A). The results showed that the volume of tumor in elevated OTUB1 is significantly bigger compared with that in the control group, which is conversely restrained by XAV-939; while the volume of tumor in the elevated β-catenin group also is bigger than that in the control group (Fig. 8B). OTUB1 significantly promotes the growth of bladder tumors *in vivo*, while XAV-939 restrains the increasing tendency; overexpressed β-catenin also achieves the same promoting effects on bladder tumor growth (Fig. 8C). Furthermore, we further found that the expression of β-catenin, ki-67 and downstream targets are induced by overexpressed OTUB1, which is conversely inhibited by XAV-939. Interestingly, the expression of OTUB1 was not affected by XAV-939 application or overexpressed β-catenin (Fig. 8 D, E). The expression of RIPK3, MLKL and P-MLKL was also inhibited by elevated OTUB1 and β-catenin, while increased by XAV-939 application (Fig. 8E), which was consolidated with the results *in vitro*. In summary, OTUB1 deubiquitinates and stabilizes β-catenin, and accumulated β-catenin enters the nucleus and further promotes the transcription process of downstream targets, thus facilitating the tumorigenesis and cisplatin resistance of BLCA (Fig. 8F).

## Discussion

It has been known that protein ubiquitination is the most common process in protein posttranslational modification and degradation, and the precise regulation of many critical factors depends on the homeostasis between ubiquitination and deubiquitination^21^. Because of the deubiquitinating activity, OTUB1 has been identified to be involved in multiple pathophysiological processes, such as DNA damage repair, multiple cancer tumorigeneses (including several solid and non-solid tumors), various immune-related disorders and so on^9^, ^22^. It has been identified preferred to remove the K48-linked ubiquitin chain from substrate protein, which is closely involved in degradation^23^. Unlike other deubiquitinase family members, OTUB1 cleavage ubiquitin chains for c deubiquitinating function in two distinct manners: the canonical manner is to directly cleavage mono-/poly- ubiquitin chains from substrate protein depending on deubiquitinase catalytic activity; the non-canonical manner is to restrain the ubiquitin transfer from E2 ubiquitin-conjugating enzyme to E3 ubiquitin ligase to further block the ubiquitination process, which depends on the N-terminal ligation activity to E2 ubiquitin-conjugating enzyme but independent catalytic activity^24^. For example, OTUB1 restrains histone ubiquitination by interacting with E2s RNF168/UBC13 dependent on the non-canonical manner^25^. OTUB1 facilitates the occurrence and progression of breast cancer by inhibiting the ubiquitination of FOXM1 dependent on the canonical manner^12^. Furthermore, OTUB1 not only regulates the activity of E2 ubiquitin-conjugating enzymes but also is stimulated by un-charged E2s^26^. OTUB1 stabilizes E2s UBE2E1 by inhibiting its auto-ubiquitination independent of the deubiquitinase activity, and the activity of OTUB1 in removing the K48 poly-ubiquitin chain can further be stimulated by OTUB1-E2s complex in turn^27^. Previous studies have reported that the Asp88 and Cys91 sites of OTUB1 are critical for the activity of DUBs, in which Asp88 is important for its link activity to E2s ubiquitin-conjugating enzyme^28^, while Cys91 is critical for the deubiquitinase catalytic activity^29^.

Multiple studies have shown that dysregulated OTUB1 is involved in the tumorigenesis, progression and chemoresistance of several cancers through stabilizing some proteins. The relationship between OTUB1 and BLCA still is just a preliminary concept, especially in chemoresistance. The canonical Wnt/β-catenin signaling pathway has been known to be involved in multiple biological and pathological processes, and its dysregulation is related to various pathologies such as tumorigenesis, congenital disorder and degenerative diseases, etc^30^, ^31^. β-catenin is the core component in the Wnt/β-catenin signaling pathway and is responsible for signal transduction and transcriptional regulation^32^. Such an important position and function determine the complex regulatory network, β-catenin activity is precisely regulated by phosphorylation and ubiquitination. Its phosphorylation usually involves activation and degradation^33^, while ubiquitination involves the degradation and termination of signal transduction etc^34^. The currently widely accepted mechanism is that β-catenin interacts with the APC complex and is phosphorylated by GSK3β, phosphorylated β-catenin further triggers E3 ubiquitin ligase β-TRCP-mediated ubiquitination of β-catenin^35^, ^36^. In addition, several other ubiquitin ligases also were identified to be involved in the ubiquitination of β-catenin, such as TRIM33^37^, Mule^38^, jade-1^39^, Siah-1^40^ and C-cbl^41^. The complicated ubiquitination regulatory network and mechanism further expounded the core role of β-catenin in multiple pathophysiological processes and signal transduction. As important as ubiquitination, deubiquitination also is a crucial part of maintaining β-catenin homeostasis, signaling and transcriptional regulation. For example, USP4 interacts with TCF4 and further deubiquitinates it, thus restraining β-catenin dependent process of transcription^42^; USP47^43^, USP2a^44^, USP5^45^, USP20^46^ are identified to deubiquitinating and stabilizing β-catenin directly. In our study, we newly identified that OTUB1 facilitates the tumorigenesis and cisplatin resistance of BLCA through deubiquitinating and stabilizing β-catenin directly. Although the relationship between OTUB1 and β-catenin has been introduced in colorectal cancer^17^, our study further explained the more detailed interaction between OTUB1 and β-catenin, emphasizing the effects of the OTUB1/β-catenin axis in BLCA and the specific domain of interaction. Accumulating evidence suggests that certain cancer cells can undergo necroptosis under some physical or chemical stimuli, and such triggering necroptosis could be a promising alternative strategy for killing cancer cells that fail to die by apoptosis. RIPK1/RIPK3/MLKL-dependent necroptosis as a new form of cell death would be further explored to develop new anti-cancer therapies to overcome the resistance to proapoptotic chemotherapeutic agents^47^. The relationship between necroptosis and multiple cancer have been identified, but the association between necroptosis and BLCA remains unclear. Yuanhui Gao et al found that Scabertopin restrains the proliferation and invasion capacity by promoting the phosphorylation of RIPK1, RIPK3 and MLKL and the necroptosis signaling pathway^48^. More necroptosis-related risk models in BLCA further showed that the prognosis of BLCA patients was tightly associated with necroptosis^49^, ^50^. Moreover, Yonggang Wang et al identified that PKM2 inhibitor Shikonin overcomes the cisplatin resistance in BLCA by inducing necroptosis, which implied that downregulated necroptosis might facilitate the chemoresistance and progression^51^. In previous study, Josephin Koschel et al found that OTUB1 reduced K48-linked polyubiquitination of c-IAP1, thereby diminishing its degradation. In the absence of OTUB1, c-IAP1 degradation resulted in reduced K63-linked polyubiquitination and increased phosphorylation of RIPK1, RIPK1/RIPK3 necrosome formation, MLKL phosphorylation and hepatocyte death. OTUB1-deficiency induced RIPK1-dependent ERK activation and TNF production in Lm-infected hepatocytes^18^. Based on the above conclusion, we found that elevated OTUB1 restrain the necroptosis progression. As for the correlation between necroptosis and β-catenin, previous studies have shown the complicated regulatory network. Such as β-catenin signaling was identified to restrain the expression of RIPK3, P-MLKL and necroptosis in liver ischemia and reperfusion injury^19^; while β-catenin/Foxo1/TLR4 signaling was found to promote necroptosis in non-alcoholic fatty liver disease^52^.

The relationship among OTUB1, BLCA, β-catenin, and necroptosis has remained elusive. In our study, we aimed to elucidate the mechanism by which OTUB1 facilitates BLCA progression and cisplatin resistance by modulating necroptosis signaling via deubiquitinated β-catenin. Firstly, we observed a significant elevation of OTUB1 in BLCA, correlating with poor prognosis compared to other members of the OTU deubiquitinase superfamily, a conclusion further supported by clinical sample analysis. Next, we established a mice BLCA model induced by feeding 0.1% BNN in water for three months. Compared to the control group, mice in the BNN-induced group exhibited enlarged bladder volumes, particularly in the 7-12 month group. Histological and immunohistochemical analyses confirmed the development of BLCA in the BNN-induced group, with OTUB1 and ki-67 expression elevated compared to controls, suggesting OTUB1's involvement in BLCA onset and progression. Further *in vitro* experiments demonstrated that elevated OTUB1 significantly promoted BLCA proliferation and invasion. RNA-seq analysis revealed OTUB1's involvement in epithelial-mesenchymal transition and the Wnt/β-catenin signaling pathway. Through database forecast and CO-IP assay, we discovered that OTUB1 interacts with β-catenin, inhibiting its proteasome degradation and facilitating its nuclear translocation, thus promoting BLCA tumorigenesis and progression. Specific interaction fragments between OTUB1 (47-271 domain fragments) and β-catenin (1-694 domain fragments) were identified, with OTUB1's deubiquitinase catalytic activity crucial for maintaining β-catenin stabilization. Additionally, we found that XAV-939, a β-catenin inhibitor, counteracted the promoting effects of OTUB1, while elevated β-catenin rescued the effects of shOTUB1. *In vivo* experiments further validated that OTUB1 facilitates tumorigenesis and progression by stabilizing β-catenin, with XAV-939 significantly inhibiting the growth effects of elevated OTUB1. We also identified the correlation between the OTUB1/β-catenin/necroptosis axis and chemotherapy response in BLCA, demonstrating that elevated OTUB1 promotes cisplatin resistance, which is reversed by XAV-939 application.

In conclusion, our study unveils the oncogenic role of OTUB1 in BLCA and integrates the complex relationship among OTUB1, BLCA, β-catenin, and necroptosis. Targeting the OTUB1/β-catenin axis or applying XAV-939 may hold promise as a therapeutic strategy for BLCA patients. Mechanistically, OTUB1 deubiquitinates and stabilizes β-catenin, leading to transcriptional upregulation of c-myc and cyclin D1 while downregulating RIPK3 and MLKL, ultimately driving tumorigenesis and cisplatin resistance in BLCA. Clinically, the OTUB1/β-catenin/RIPK3/MLKL axis may serve as a potential biomarker for predicting cisplatin resistance in BLCA.

## Supplementary Material

Supplementary figures.
