# Supplementary material for: Target deubiquitinase OTUB1 as a therapeatic strategy for BLCA via β-catenin/necroptosis signal pathway
Source: Int J Biol Sci. 2024 Jul 2;20(10):3784–801. doi: 10.7150/ijbs.94013 (PMC11302878; doi:10.7150/ijbs.94013)
Supplement: Supplementary file 1 — Supplementary figures. [file ijbsv20p3784s1.pdf]

Supplemental figure legends

Supplementary Figure 1. Supplementary paired BLCA tissue western blot. The protein expression of OTUB1 in BLCA tissue (T) is higher compared with normal bladder tissue (N).

Supplementary Figure 2. OTUB1 expression is positively related to β-catenin expression. A. The expression of OTUB1 and β-catenin in BLCA tissue and normal bladder tissue is based on the Human Protein Atlas. B. The expression of OTUB1 and β-catenin in BLCA tissue and paired bladder tissue from our hospital. C, D. Quantification of OTUB1 and β-catenin expression in BLCA and paired bladder tissues. E. Correlation study of OTUB1 and β-catenin in BLCA. Statistical analyses were performed with the $\chi 2$ test. R: The Pearson correlation coefficient.

Supplementary Figure 3. OTUB1 is associated with the Wnt-β-catenin signaling pathway. A. Relative expression of OTUB1 following shOTUB1 lentivirus transfection by western blot and qRT-PCR. B. The relationship between OTUB1 and β-catenin was analyzed by the gene MANIA online database. C. The correlation between OTUB1 and β-catenin is based on the GEPIA database. D, E. Gene Ontology and KEGG annotations of differential genes involved in biological processes, molecular function and cellular compartments. F. GSEA of RNA-seq data revealed that OTUB1 target genes were involved in EMT signaling pathway.

Supplementary Figure 4. The relationship and regulation between OTUB1 and other isoforms. A. The downstream molecular expression about OTUB1, C91S, and D88A by western blot. B. Relative expression of

OTUB1 following OTUB1, C91S, and D88A plasmid transfection by qRT-PCR. C. Relative expression of β-catenin following OTUB1, C91S, and D88A plasmid transfection by qRT-PCR. D. Relative expression of CDK4 following OTUB1, C91S, and D88A plasmid transfection by qRT-PCR. E. Relative expression of cyclin D1 following OTUB1, C91S, and D88A plasmid transfection by qRT-PCR. F. Relative expression of RIPK3 following OTUB1, C91S, and D88A plasmid transfection by qRT-PCR. G. Relative expression of MLKL following OTUB1, C91S, and D88A plasmid transfection by qRT-PCR. H. Relative expression of C91S following shOTUB1 lentivirus transfection by western blot and qRT-PCR. I. VRelative expression of D88A following shOTUB1 lentivirus transfection by western blot and qRT-PCR.

Supplementary Figure 1

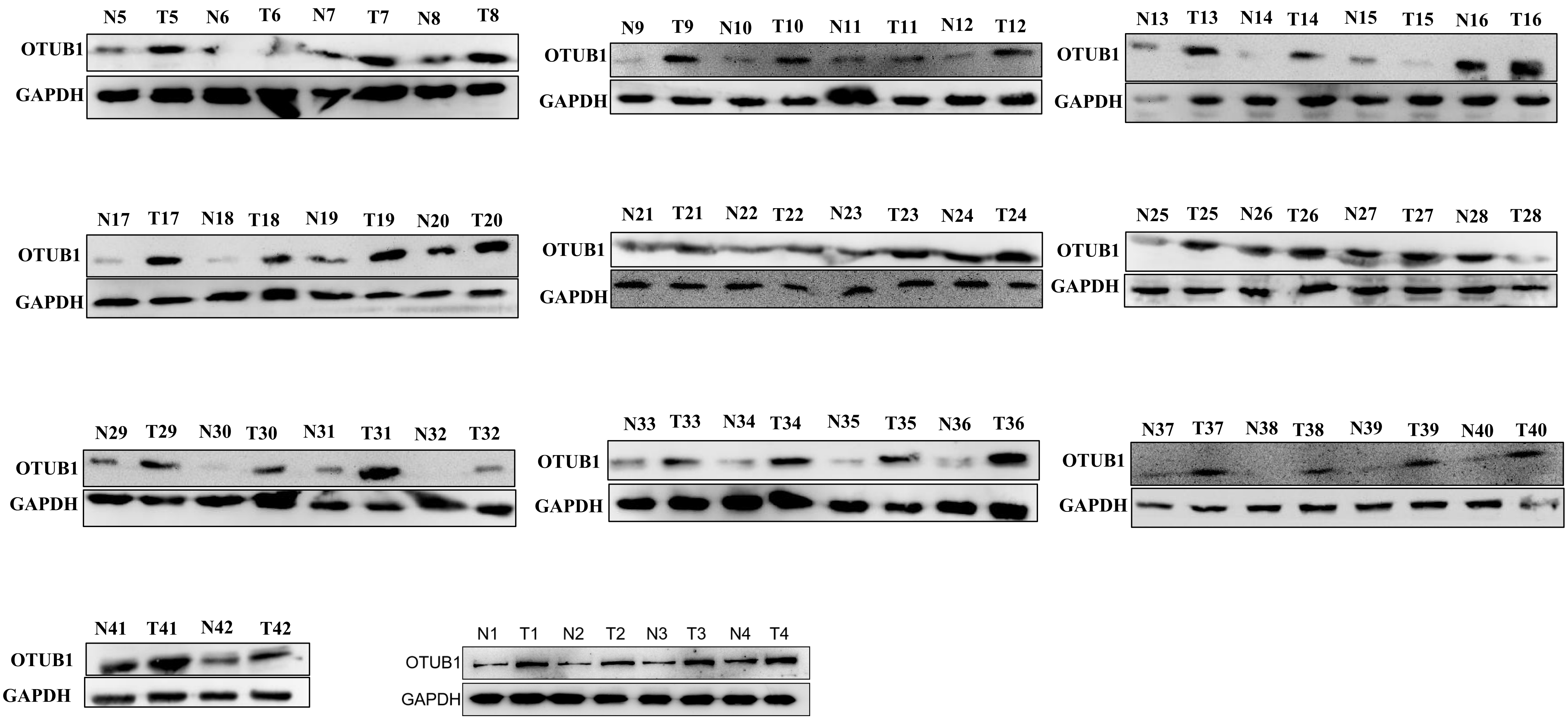

A

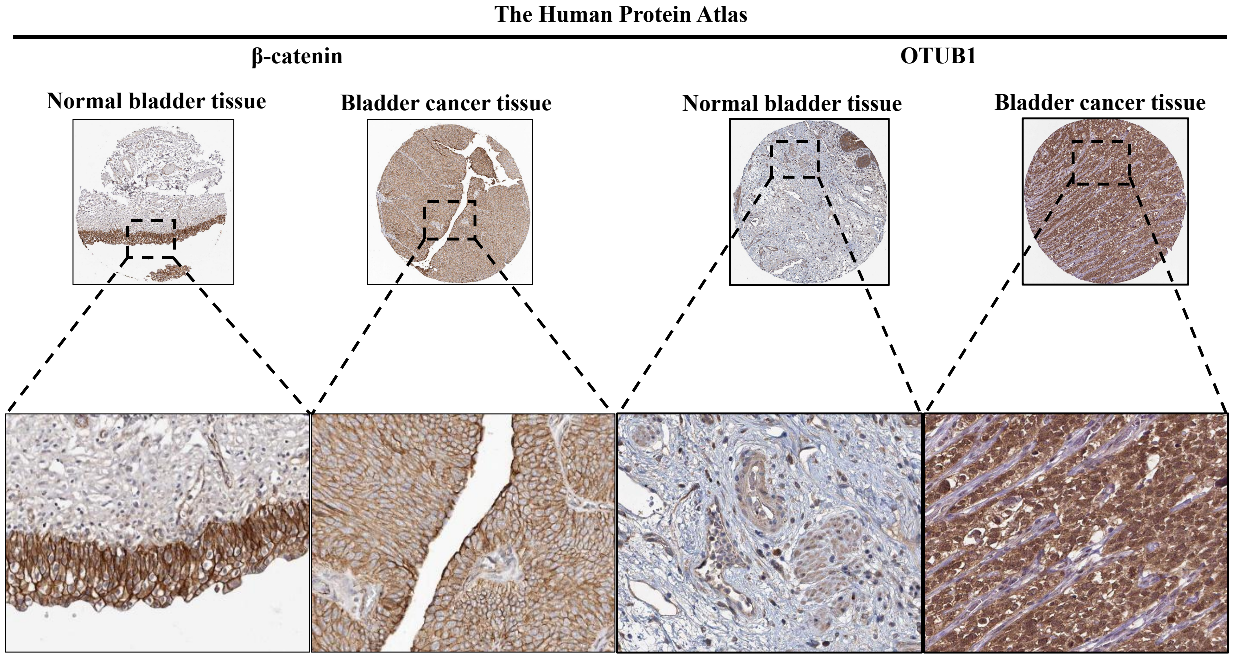

B

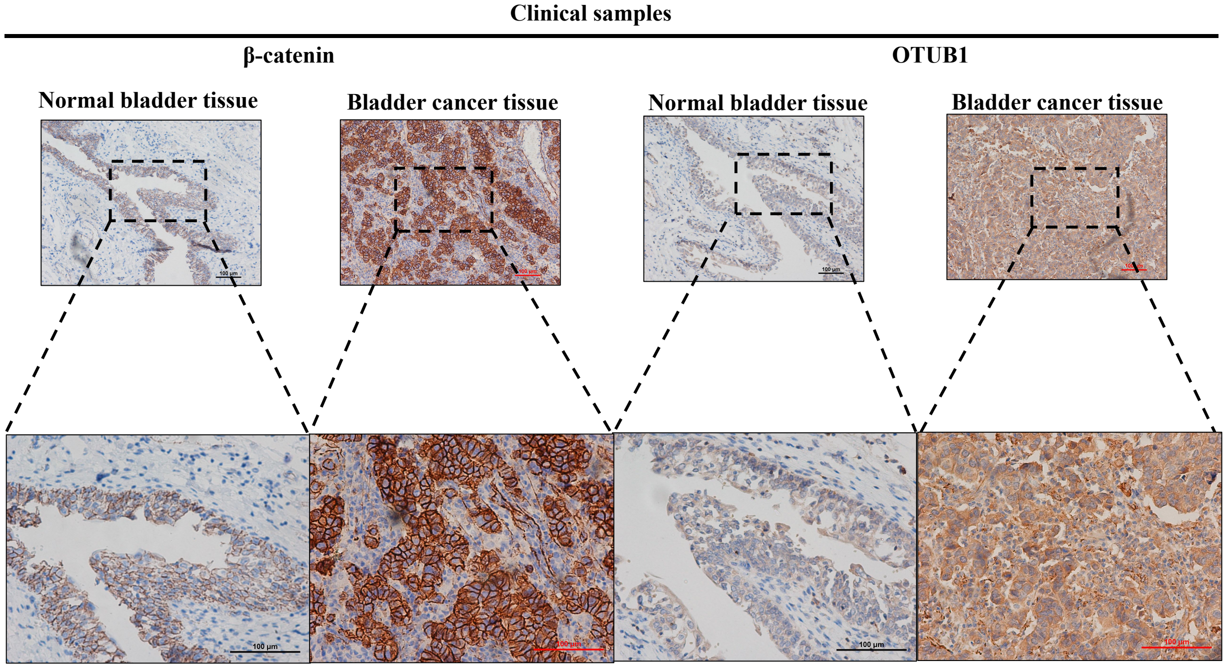

C

OTUB1	High	Low	Total
Normal Bladder	11	31	42
Bladder Cancer	88	17	105

D

β-catenin	High	Low	Total
Normal Bladder	4	38	42
Bladder Cancer	82	23	105

E

	β-catenin High	β-catenin Low	Total
OTUB1 High	76	12	88
OTUB1 Low	6	11	17
Total	82	23	105

$P < 0.001$; R=0.455

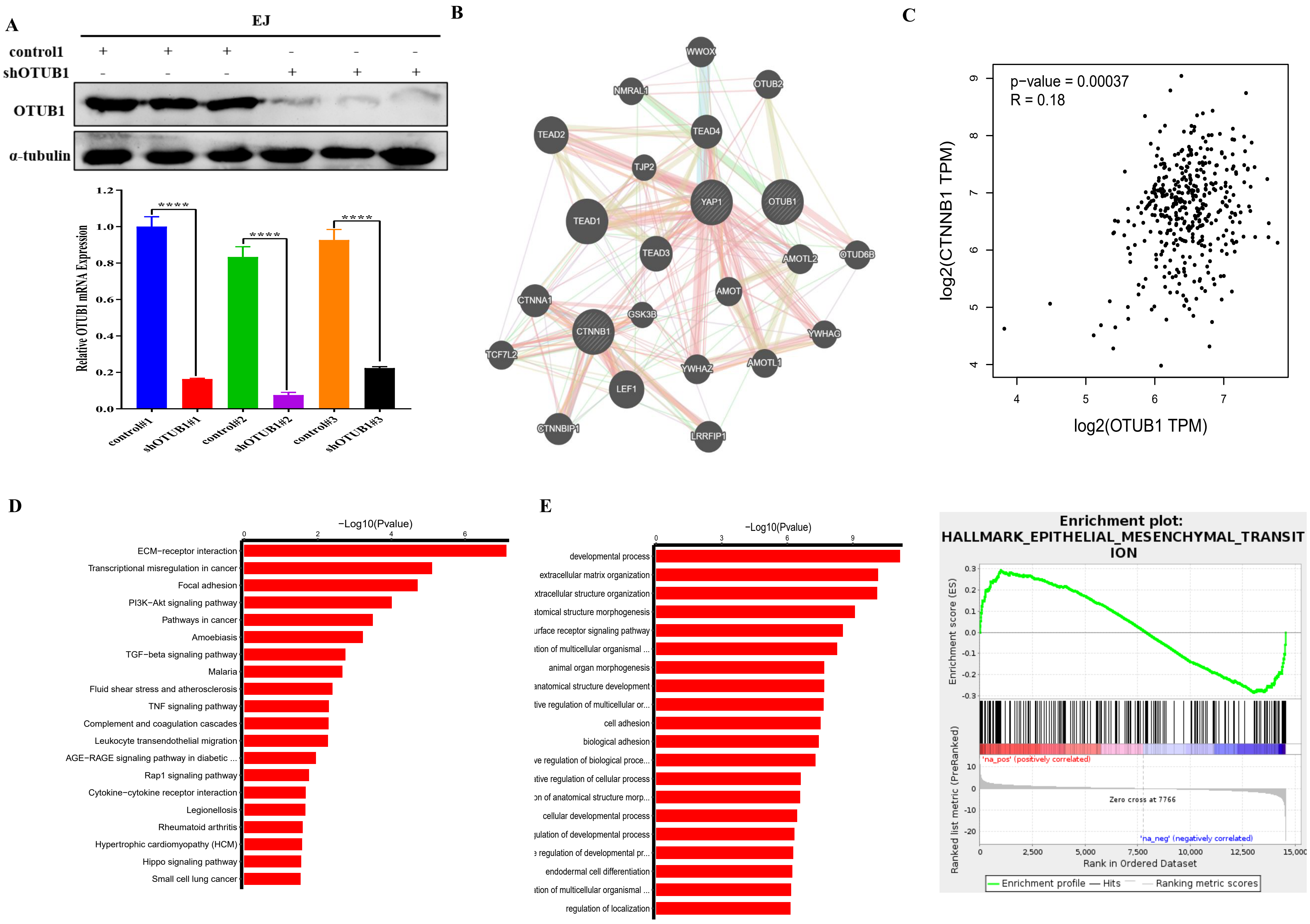

A
EJ
control1
shOTUB1
OTUB1
α-tubulin
Relative OTUB1 mRNA Expression
****
control#1
shOTUB1#1
control#2
shOTUB1#2
control#3
shOTUB1#3
B
WWOX
NMRAL1
OTUB2
TEAD2
TEAD4
TJP2
YAP1
OTUB1
TEAD1
TEAD3
AMOTL2
OTUD6B
AMOT
CTNNA1
GSK3B
CTNNB1
TCF7L2
YWHAG
YWHAZ
AMOTL1
LEF1
CTNNBIP1
LRRFIP1
C
p−value = 0.00037
R = 0.18
log2(CTNNB1 TPM)
log2(OTUB1 TPM)
D
−Log10(Pvalue)
ECM−receptor interaction
Transcriptional misregulation in cancer
Focal adhesion
PI3K−Akt signaling pathway
Pathways in cancer
Amoebiasis
TGF−beta signaling pathway
Malaria
Fluid shear stress and atherosclerosis
TNF signaling pathway
Complement and coagulation cascades
Leukocyte transendothelial migration
AGE−RAGE signaling pathway in diabetic ...
Rap1 signaling pathway
Cytokine−cytokine receptor interaction
Legionellosis
Rheumatoid arthritis
Hypertrophic cardiomyopathy (HCM)
Hippo signaling pathway
Small cell lung cancer
E
−Log10(Pvalue)
developmental process
extracellular matrix organization
xtracellular structure organization
atomical structure morphogenesis
urface receptor signaling pathway
ation of multicellular organismal ...
animal organ morphogenesis
anatomical structure development
tive regulation of multicellular or...
cell adhesion
biological adhesion
ve regulation of biological proce...
itive regulation of cellular process
on of anatomical structure morp...
cellular developmental process
julation of developmental process
e regulation of developmental pr...
endodermal cell differentiation
ation of multicellular organismal ...
regulation of localization
Enrichment plot:
HALLMARK_EPITHELIAL_MESENCHYMAL_TRANSITION
Enrichment score (ES)
Ranked list metric (PreRanked)
'na_pos' (positively correlated)
Zero cross at 7766
'na_neg' (negatively correlated)
Rank in Ordered Dataset
Enrichment profile — Hits — Ranking metric scores

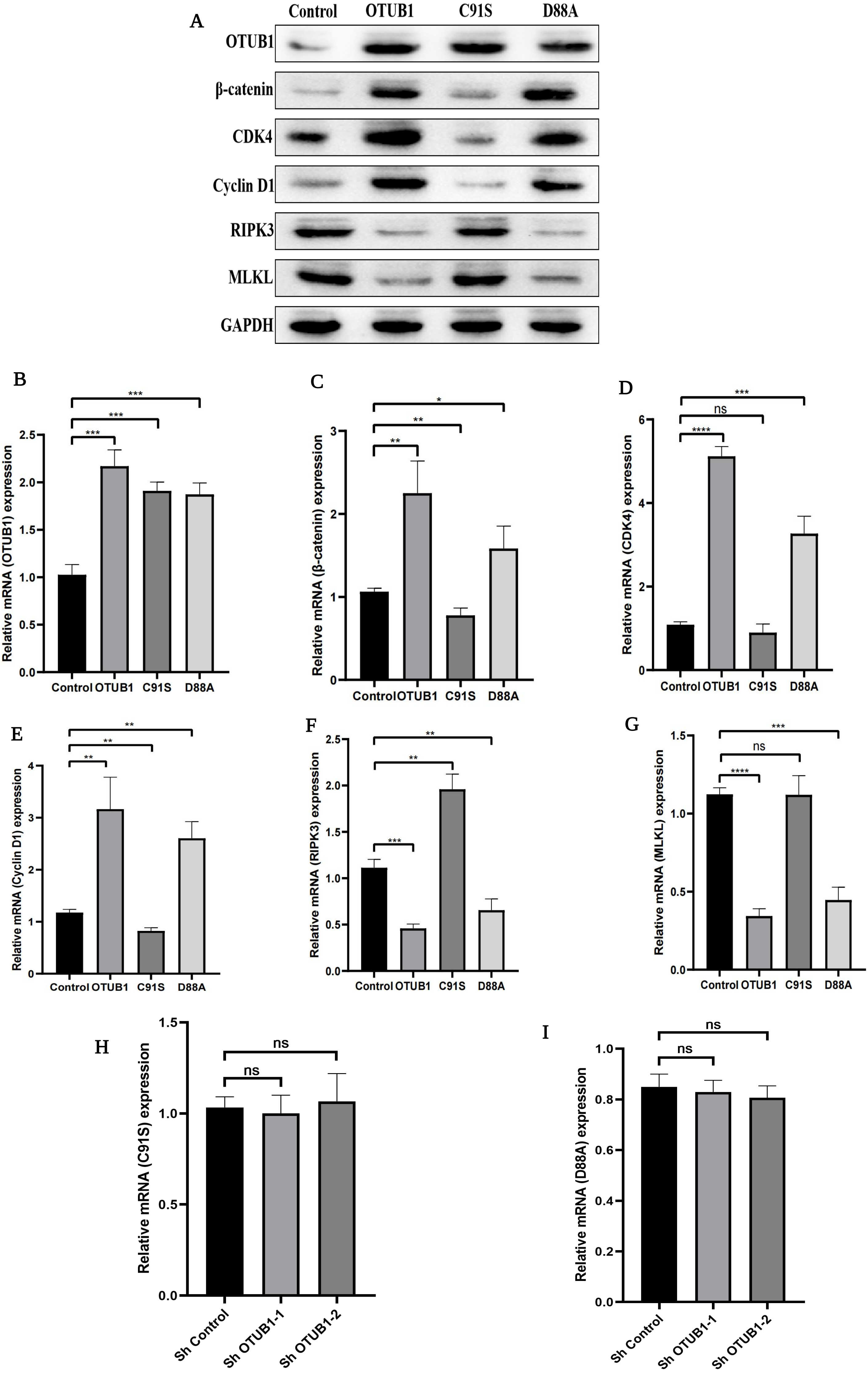
A
Control
OTUB1
C91S
D88A
OTUB1
β-catenin
CDK4
Cyclin D1
RIPK3
MLKL
GAPDH
B
Relative mRNA (OTUB1) expression
C
Relative mRNA (β-catenin) expression
D
Relative mRNA (CDK4) expression
E
Relative mRNA (Cyclin D1) expression
F
Relative mRNA (RIPK3) expression
G
Relative mRNA (MLKL) expression
H
Relative mRNA (C91S) expression
Sh Control
Sh OTUB1-1
Sh OTUB1-2
I
Relative mRNA (D88A) expression
ns
